# The immunoproteome and multimorbidity: A Mendelian randomization study

**DOI:** 10.1126/sciadv.adz7117

**Published:** 2026-05-20

**Authors:** Nikita Hukerikar, Aroon D. Hingorani, Sandesh Chopade, Arjen J. Cupido, Folkert W. Asselbergs, Chris Finan, A. Floriaan Schmidt

**Affiliations:** ^1^ Institute of Health Informatics, Faculty of Population Health Sciences, University College London, 222 Euston Road, London NW1 2DA, UK.; ^2^ Institute of Cardiovascular Science, Faculty of Population Health, University College London, 69-75 Chenies Mews, London WC1E 6HX, UK.; ^3^ UCL British Heart Foundation Centre of Excellence, 69-75 Chenies Mews, London WC1E 6HX, UK.; ^4^ Department of Cardiology, Amsterdam Cardiovascular Sciences, Amsterdam University Medical Centres, University of Amsterdam, Amsterdam UMC, locatie AMC Postbus 22660, 1100 DD Amsterdam-Zuidoost, Netherlands.; ^5^ Department of Internal Medicine, Amsterdam University Medical Centres, University of Amsterdam, Amsterdam UMC, locatie AMC Postbus 22660, 1100 DD Amsterdam-Zuidoost, Netherlands.; ^6^ Department of Internal Medicine, Tergooi MC, Laan van Tergooi 2, 1212 VG Hilversum, Netherlands.; ^7^ National Institute for Health Research University College London Hospitals Biomedical Research Centre, UCLH/UCL Joint Research Office, 250 Euston Road, London NW1 2PG, UK.

## Abstract

Multimorbidity presents challenges for research and health care. We investigated how immune-proteins influence multiple diseases to identify shared mechanisms and therapeutic opportunities. Using eight large plasma proteome GWAS, we identified cis-acting variants for 151 immune-proteins and applied cis-Mendelian randomization to assess associations with 64 diseases and biomarkers. Protein-disease communities were derived using a knowledge graph integrating multiplicity-corrected associations, druggability, indications, and tissue-trait associations. Replication was sought in independent pQTLs, with colocalization applied to replicated signals. Immune-proteins were frequently implicated in coronary heart disease, venous thromboembolism, atrial fibrillation, type 2 diabetes, and Parkinson’s disease. Seven protein communities mapped to pathways including ficolin binding and antimicrobial peptides, enriched for proteins influencing multiple diseases. Several associations replicated with colocalization support, including APOE, CD163, and IL-6R across neurodegenerative, cardiometabolic, and inflammatory traits. We found strong genetic support for druggable immune-proteins with pleiotropic effects across common diseases, highlighting opportunities for indication expansion and therapeutic development.

## Teaser

Genetic insights show how immune-related proteins affect multiple diseases, revealing shared pathways and treatment opportunities.

## INTRODUCTION

The accumulation of multiple diseases through the life course of an individual means that older individuals frequently suffer several diseases concurrently, referred to as multimorbidity. Diseases without a shared cause co-occur at rates consistent with their individual frequencies (*1*). Diseases that share common causes should also co-occur, but at a frequency that exceeds that predicted on the assumption of their independence (disease clustering) (*1*). Using routine health care data from 4 million people in the National Health Service in England, we previously showed that nonrandom disease clusters are common (*2*), raising a question about the underlying mechanism.

Theory and empirical evidence from genome-wide association studies (GWAS) already indicate that many different diseases must share common genetic predisposition (pleiotropy) (*3*–*5*). This implies the same genes, and their encoded proteins could be critical to the pathogenesis of a range of disorders. Around a quarter of proteins are druggable, with this proportion expected to increase as new therapeutic modalities expand the range of tractable targets. Additionally, 98% of all drug targets are proteins (*6*), so GWAS findings have potential therapeutic implications.

Inflammation and inflammatory cytokines have been implicated in immune-mediated inflammatory diseases (IMIDs) involving the joints (rheumatoid and seronegative arthritis), skin (psoriasis and atopic dermatitis), gut (inflammatory bowel disease), and nervous system (multiple sclerosis). For example, monoclonal antibodies targeting tumor necrosis factor (TNF) are used in rheumatoid, juvenile, and psoriatic arthritis as well as in Crohn’s disease and ulcerative colitis; antibodies targeting interleukin-12 (IL-12) and IL-23 signaling are used in psoriasis and ulcerative colitis. There is also evidence from genetic studies and clinical trials that inflammatory cytokines may contribute to diseases that were not traditionally considered to be inflammatory, including cardiometabolic disease (*7*, *8*), cancer (*9*), and neurodegenerative disease (*10*). For example, genetic evidence at the IL-6 receptor (IL-6R) locus implicated IL signaling in coronary heart disease (CHD), and the CANTOS trial subsequently demonstrated that inhibition of IL-1β, an upstream mediator of IL-6 signaling, reduced recurrent cardiovascular events in patients with established CHD (*9*). Therefore, immune-related proteins and the related inflammatory processes may form a common and important pathological link driving distinct patterns of multimorbidity.

Cytokines form a subset of immune-related proteins [“the immunoproteome”; (*11*)] comprising of circulating ILs, chemokines, complement components, and their soluble receptors. Together with their membrane-bound receptors and intracellular signaling pathways involving protein kinases, these proteins provide hundreds of potential therapeutic targets not just for IMIDs but also a wide range of other common diseases (*11*–*13*) contributing to multimorbidity.

GWAS have revealed associations between genetic variants and circulating plasma proteins involved in the immune response, as well as with common diseases that contribute to multimorbidity. When genetic variants located near a gene (acting in cis) are robustly associated with protein levels, they can be used as instruments to test whether genetically predicted differences in that protein are associated with disease risk. Evidence of such a genetic dose-response relationship supports a putative disease-modifying role for the protein (*14*–*16*). This type of analysis is referred to as cis-MR, a form of instrumental variable analysis that attempts to identify putative causal effects of an exposure on an outcome. Because genetic variation is randomly allocated during gametogenesis, and disease status does not influence heritable genetic changes, genetic analyses such as cis-MR are protected from confounding and reverse causation, allowing inference on the potential role of a protein in development of a disease (*17*). To strengthen the evidence for a protein-disease effect, complementary genetic methods such as colocalization are often used as a post-MR step. Colocalization estimates if the genetic signals for a protein and a disease arise from the same underlying causal variant, thereby helping to prioritize putative drug targets. The presence of genetic evidence has become increasingly relevant for the identification and prioritization of drug targets for (early) drug development. Retrospective analyses have shown that drug candidates supported by genetic evidence are approximately 2.6 times more likely to achieve regulatory approval (*18*, *19*).

In this study, we performed cis-MR to identify immune proteins with genetic evidence consistent with a potential disease risk modifying effect, related to diseases contributing to multimorbidity, as well as to disease risk factors such as blood pressure and circulating cholesterol fractions. We subsequently identified potential therapeutically interesting targets using a combination of direct replication, graph-based learning, and evaluating of directional consistency. Specifically, genetic instruments indexing circulating plasma proteins were sourced from five independent GWAS of 151 immune proteins, measured in between 1328 and 35,559 participants. The multiplicity corrected associations with 50 clinically relevant diseases and 14 disease biomarkers were then prioritized through consideration of information on biological pathways, tissue expression, tissue-trait association and protein druggability. The associations and annotations were encoded into a knowledge graph, which was used to identify communities of interrelated immune proteins relevant for multiple diseases. We additionally sought to differentiate prioritized proteins with consistently harmful or protective effects, compared to proteins with a mixed effect profile. Last, we conducted colocalization and replication analyses on the Mendelian randomization (MR)–associated proteins to identify a subset of proteins with the strongest genetic evidence for causality and association with the diseases and biomarkers.

## RESULTS

There were sufficient genetic instruments to conduct MR analysis for 151 immune proteins, 129 of which were associated with at least one disease or biomarker and were associated with a median number of 8 diseases or biomarkers [quartile 1 (Q1), 4; and Q3, 13]; see table S1. The 10 most pleiotropic proteins (table S2) were IL-17RD, CCL17, FCGR2B, IL-6R, RNASE6, SPON2, C4B, CXCL11, NMB, and ATRN. These proteins affected not only IMIDs but also metabolic traits, stroke, and certain cancers (table S2). Of these 129 proteins, 73 were targeted by approved or developmental medicines, 40 were druggable, and 16 were not yet druggable (table S3).

**Fig. 1.**
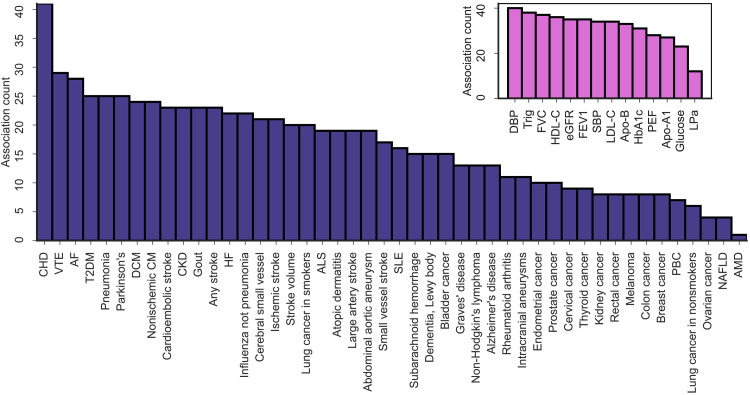
The number of proteins associating with a disease or disease-biomarker based on cis-MR analysis of 151 proteins. The *y* axis represents the number of MR association with a *P* value smaller than the 5.16 × 10^−6^ multiplicity corrected threshold. Purple bars indicate disease outcomes, and pink bars represent disease biomarkers. The MR analyses sourced GWAS on plasma protein value from Said *et al.* (*66*) (*n* = 575,531), Ferkingstad *et al.* (*67*) (*n* = 35,559), Gilly *et al.* (*68*) (*n* = 1328), Sun *et al.* (*69*) (*n* = 3301), Folkersen *et al.* (*70*) (*n* = 30,931), Yang *et al.* (*71*) (*n* = 835), Yao *et al.* (*72*) (*n* = 6861), and Ahola-Olli *et al.* (*73*) (*n* = 8293). For the source GWAS on disease and disease biomarkers, please refer to table S20. The numerical data underlying this illustration are available in table S1. AF, atrial fibrillation; ALS, amyotrophic lateral sclerosis; AMD, age-related macular degeneration; Apo-A1, apolipoprotein A1; Apo-B, apolipoprotein B; CHD, coronary heart disease; CKD, chronic kidney disease; DBP, diastolic blood pressure; DCM, dilated cardiomyopathy; eGFR, estimated glomerular filtration rate; FEV1, forced expiratory volume; FVC, forced vital capacity; HDL-C, high-density lipoprotein cholesterol; HF, heart failure; HbA1c, hemoglobin A1c; LDL-C, low-density lipoprotein cholesterol; LPa, lipoprotein (a); NAFLD, nonalcoholic fatty liver disease; nonischemic CM, nonischemic cardiomyopathy; PBC, primary biliary cirrhosis; PEF, peak expiratory flow;SBP, systolic blood pressure; SLE, systemic lupus erythematosus; T2DM, type 2 diabetes mellitus; Trig, triglycerides; VTE, venous thromboembolism.

In general, we found that immune-related proteins were associated with the risk of CHD (41 associated proteins), venous thromboembolism (VTE) (29), atrial fibrillation (AF) (28), as well as type 2 diabetes mellitus (T2DM) (25), pneumonia (25), and Parkinson’s disease (25), Fig. 1.

### Proteins with a predominantly harmful or beneficial disease effect

**Fig. 2.**
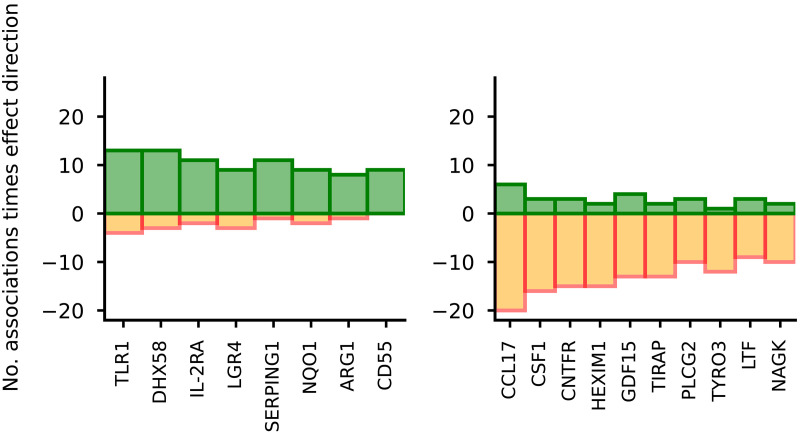
Immune-related plasma proteins with predominantly harmful or beneficial effects on 64 diseases and disease biomarkers. The effect estimates are based on cis-MR analysis with a *P* value smaller than a multiplicity corrected threshold of 5.16 × 10^–6^. The MR analyses sourced GWAS on plasma protein value from Said *et al.* (*66*) (*n* = 575,531), Ferkingstad *et al.* (*67*) (*n* = 35,559), Gilly *et al.* (*68*) (*n* = 1328), Sun *et al.* (*69*) (*n* = 3301), Folkersen *et al.* (*70*) (*n* = 30,931), Yang *et al.* (*71*) (*n* = 835), Yao *et al.* (*72*) (*n* = 6861), and Ahola-Olli *et al.* (*73*) (*n* = 8293). For the source GWAS on disease and disease biomarkers, please refer to table S20. The numerical data underlying this illustration are available in table S4. (**Left**) Proteins where at least 75% of associations have a beneficial effect direction. (**Right**) Proteins where at least 75% of associations have a harmful effect direction.

We explored proteins where an increase in plasma level had a consistent (i.e., 75% of significant associations) beneficial or harmful effect on diseases or disease biomarkers, based on the previously established beneficial effect directions (table S4, Fig. 2, and fig. S1). This analysis highlighted higher values of Toll-like receptor 1 (TLR1), DHX58, IL-2RA, LGR4, SERPING1, NQO1, ARG1, and CD55 as consistently protective and higher levels of CCL17, CSF1, CNTFR, HEXIM1, GDF15, TIRAP, PLCG2, TYRO3, LTF, and NAGK as consistently harmful across disease and biomarker traits. CCL17, one of the consistently harmful proteins, was also one of the top 10 most pleiotropic. Proteins within the consistent direction set (either harmful or beneficial) were enriched for Reactome pathways involved in TLR cascades and class I major histocompatibility complex (MHC) antigen presentation (table S5).

### Exploring pleiotropic effects of immune-related proteins

**Fig. 3.**
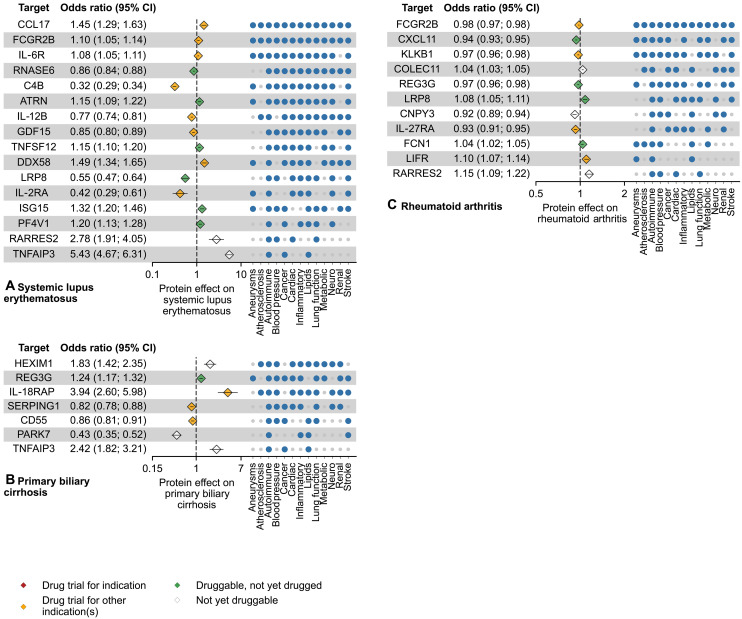
Mendelian randomisation estimates for immune-proteins associated with immune-mediated inflammatory diseases. The effect estimates are based on cis-MR analysis with a *P* value smaller than a multiplicity corrected threshold of 5.16 × 10^–6^. The MR analyses sourced GWAS on plasma protein value from Said *et al.* (*66*) (*n* = 575,531), Ferkingstad *et al.* (*67*) (*n* = 35,559), Gilly *et al.* (*68*) (*n* = 1328), Sun *et al.* (*69*) (*n* = 3301), Folkersen *et al.* (*70*) (*n* = 30,931), Yang *et al.* (*71*) (*n* = 835), Yao *et al.* (*72*) (*n* = 6861), and Ahola-Olli *et al.* (*73*) (*n* = 8293). For the source GWAS on disease and disease biomarkers, please refer to table S20. The numerical data underlying this illustration are available in table S6. The effect direction is based on a unit increase in protein concentration. Incidence matrix indicates other disease groups, in which at least one outcome is associated with a protein. Marker colors on plot indicate the drug trial status of the protein as a target as per TrialTrove (*56*) and ChEMBL (*55*). (**A**), (**B**), and (**C**) show associations for systemic lupus erythematosus, primary biliary cirrhosis and rheumatoid arthritis respectively. CI, confidence interval.

**Fig. 4.**
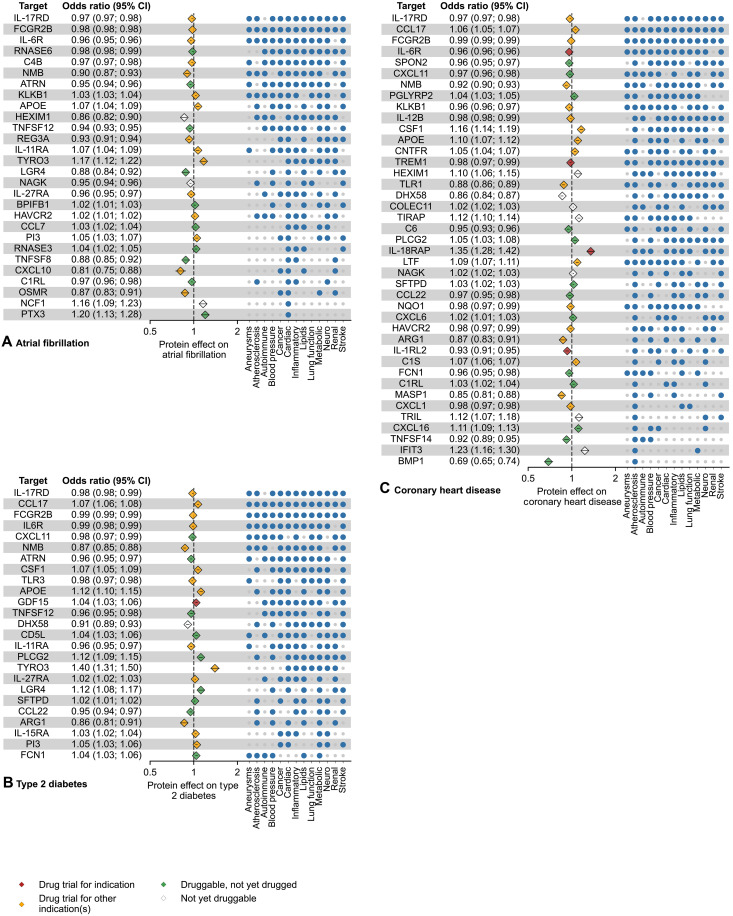
Mendelian randomisation estimates for immune-proteins associated with cardiometabolic diseases. The effect estimates are based on cis*-*MR analysis with a *P* value smaller than a multiplicity corrected threshold of 5.16 × 10^–6^. The MR analyses sourced GWAS on plasma protein value from Said *et al.* (*66*) (*n* = 575,531), Ferkingstad *et al.* (*67*) (*n* = 35,559), Gilly *et al.* (*68*) (*n* = 1328), Sun *et al.* (*69*) (*n* = 3301), Folkersen *et al.* (*70*) (*n* = 30,931), Yang *et al.* (*71*) (*n* = 835), Yao *et al.* (*72*) (*n* = 6861), and Ahola-Olli *et al.* (*73*) (*n* = 8293). For the source GWAS on disease and disease biomarkers, please refer to table S20. The numerical data underlying this illustration are available in table S7. The effect direction is based on a unit increase in protein concentration. Incidence matrix indicates other disease groups, in which at least one outcome is associated with a protein. Marker colors on plot indicate the drug trial status of the protein as a target as per TrialTrove (*56*) and ChEMBL (*55*). (**A**), (**B**), and (**C**) show associations for atrial fibrillation, type 2 diabetes and coronary heart disease respectively. CI, confidence interval.

**Fig. 5.**
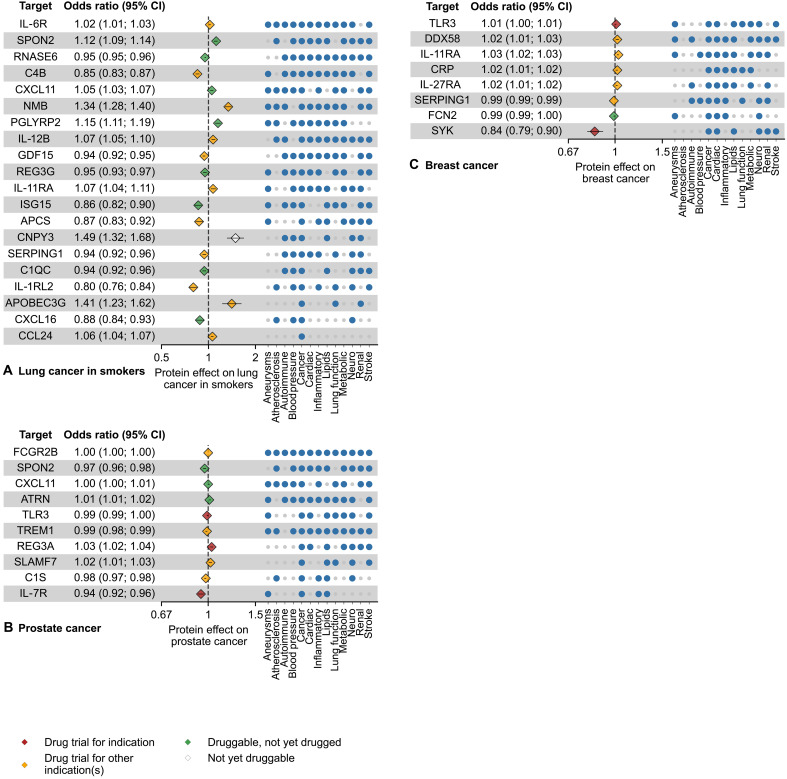
Mendelian randomisation estimates for immune-proteins associated with cancers. The effect estimates are based on cis*-*MR analysis with a *P* value smaller than a multiplicity corrected threshold of 5.16 × 10^–6^. The MR analyses sourced GWAS on plasma protein value from Said *et al.* (*66*) (*n* = 575,531), Ferkingstad *et al.* (*67*) (*n* = 35,559), Gilly *et al.* (*68*) (*n* = 1328), Sun *et al.* (*69*) (*n* = 3301), Folkersen *et al.* (*70*) (*n* = 30,931), Yang *et al.* (*71*) (*n* = 835), Yao *et al.* (*72*) (*n* = 6861), and Ahola-Olli *et al.* (*73*) (*n* = 8293). For the source GWAS on disease and disease biomarkers, please refer to table S20. The numerical data underlying this illustration are available in table S8. The effect direction is based on a unit increase in protein concentration. Incidence matrix indicates other disease groups, in which at least one outcome is associated with a protein. Marker colors on plot indicate the drug trial status of the protein as a target as per TrialTrove (*56*) and ChEMBL (*55*). (**A**), (**B**), and (**C**) show associations for lung cancer in smokers, prostate cancer and breast cancer respectively. CI, confidence interval.

Figures 3 to 5 and fig. S1 (tables S6 to S9) illustrate the individual significant protein effects on four the disease groups: IMIDs, cardiometabolic diseases, cancers, and neurological diseases. Information on clinical trials for all the targets in this study sourced from the TrialTrove database is presented in table S7.

In the IMID group [systemic lupus erythematosus (SLE), rheumatoid arthritis (RA), primary biliary cirrhosis (PBC)], 16 proteins affected SLE risk including 14 druggable targets. These proteins also frequently associated with lipids (15 protein associations), cancers (13), cardiac outcomes (13), inflammatory diseases (13), and stroke (13) (Fig. 3 and table S6). For RA, we identified 11 proteins, 8 of which were druggable. These proteins additionally associated with blood pressure traits (8 proteins), cancers (7), cardiac traits (7), and lipid levels (7). We identified seven proteins affecting PBC, including four druggable candidates. These PBC-related proteins showed pleiotropic effects on blood pressure, other inflammatory diseases, lipid levels, and lung function (five protein associations each). The IMID-related proteins included 15 proteins that have been studied as targets at any human clinical trial phase, including FGCR2B associated with both SLE and RA, REG3G associated with both RA and PBC, and LRP8 associated with both SLE and RA, presenting potential repurposing indications for IMIDs. REG3G, which has not yet been investigated at trial stages, had associations with both RA and PBC.

The results for AF, CHD, and T2DM are presented in Fig. 4 (table S7). The 28 proteins associated with AF were frequently associated with lipids (21 protein associations), metabolic traits (21), inflammatory diseases (18), and neurological diseases (18) and included 25 druggable targets. The 41 proteins associated with CHD were frequently associated with blood pressure (29 protein associations), inflammatory diseases (29), and lipids (28). Thirty-four of these proteins were druggable, including IL-6R that is the target of tocilizumab for treatment of RA and is being trialed as a target for CHD. The 25 proteins associated with T2DM were also frequently associated with lipid levels (22 protein associations), cardiac outcomes (21), inflammatory disease (19), stroke (19), cancer (18), and neurological diseases (18) and included 24 druggable targets. The cardiometabolic related proteins included 51 proteins that have been studied as targets at any human clinical trial phase, including IL-17RD where elevated levels were associated with a with a decreasing risk of AF, CHD, and T2DM; FCGR2B with a decreasing effect on all three of AF, CHD, and T2DM; CSF1 and CCL17 (with concordantly harmful effects across diseases and biomarkers), and a harmful effect on both CHD and T2DM and IL-6R with a protective effect on all three diseases, with a potential for drug repurposing. TNFSF12, a protein that has not yet been targeted in a trial, had a risk decreasing effect on all three diseases.

In the cancer group (Fig. 5 and table S8), we identified associations with lung cancer for 20 proteins, of which 19 were druggable. These proteins were also frequently associated with blood pressure (15 protein associations), inflammatory diseases (14), and renal diseases (14). The 8 proteins associated with breast cancer were frequently associated with cardiac traits (8 proteins), inflammatory disease (6), lipid levels (6), and neurological disease (6), and all 8 of these proteins were druggable. The 10 proteins associated with prostate cancer were also frequently associated with lipid levels (9), neurological diseases (8), and stroke (8) and were all druggable targets. Among the proteins associated with cancers in this group, 25 have already been trialed for various cancers at human clinical stages, including TLR3 trialed for treatment of both breast cancer and prostate cancer, IL-11RA increasing risk of both lung cancer in smokers and breast cancer, and SERPING1 (with concordantly protective effects across diseases and biomarkers) with a decreasing risk effect on both lung cancer in smokers and breast cancer. CXCL11, a druggable protein, also had associations with prostate cancer and lung cancer in smokers.

Last, in the neurological diseases group (fig. S2 and table S9), the 12 proteins associated with Alzheimer’s disease were frequently associated with lipid levels (10 protein associations) and stroke (8) and included 10 druggable proteins. Twenty-five proteins were associated with Parkinson’s disease and were also frequently associated with cardiac traits (19), stroke (19), inflammatory diseases (18), and cancer (17), including 22 druggable targets. The 19 amyotrophic lateral sclerosis (ALS)–associated proteins were also frequently associated with cardiac traits (15), lipids (15), inflammatory diseases (14), and stroke (14) including 17 druggable proteins. The 15 proteins associated with Lewy body dementia, all of which were druggable, also frequently associated with lipids (13), stroke (13), cancers (12), and metabolic diseases (12). Of the proteins in this group, 64 were druggable. These included IL-6R that increased risk of Parkinson’s disease, SPON2 that increased risk of Alzheimer’s disease but decreased risk of ALS, and CCL17 that increased risk of both ALS and Lewy body dementia.

### Protein prioritization using a knowledge graph

**Fig. 6.**
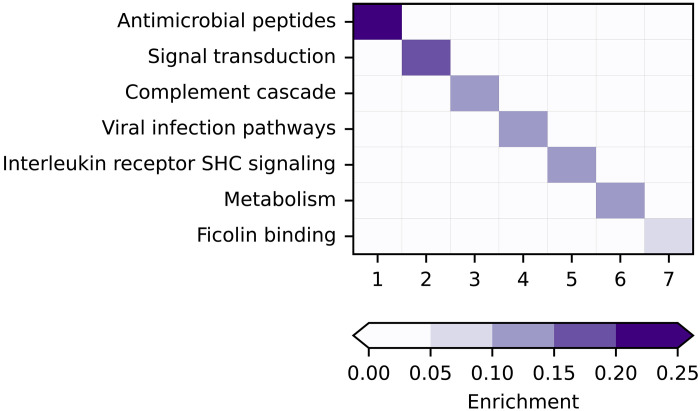
Enriched biological pathways based on community protein membership. Enrichment was calculated by comparing the proportion of proteins in a community that are members of a pathway to the proportion of proteins outside the community that are members of the same pathway. The Wald statistic was used to find pathways with statistically significant proportion differences (*P* < 0.05). The pathway with the highest enrichment value for each community is shown in the figure. If the most enriched pathway was too broad (e.g., “disease”), then the second most enriched pathway was chosen, if available. The data underlying this graph are in table S13.

**Fig. 7.**
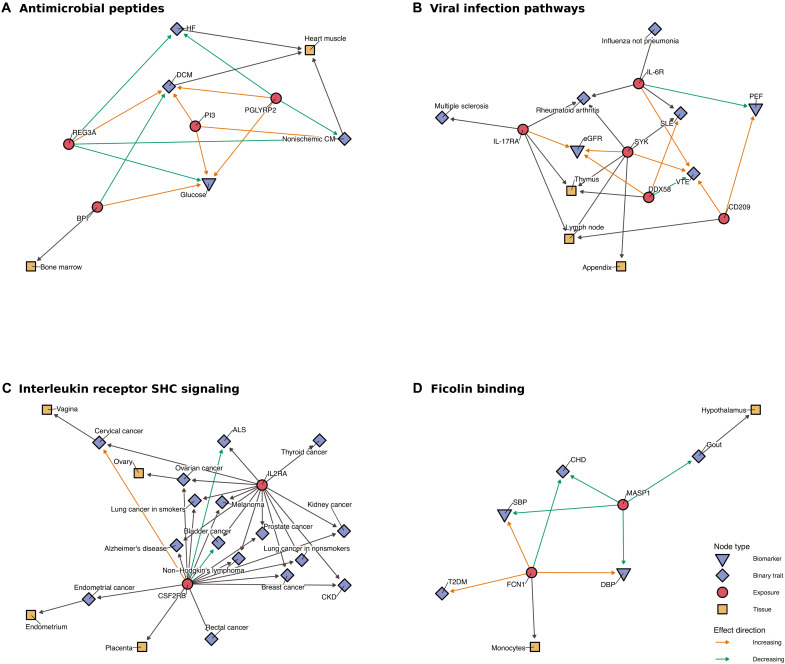
Proteins in enriched pathways for graph communities. Orange and green arcs represent significant MR arcs between protein and disease/biomarker nodes. Black arcs between protein and disease/biomarker nodes represent a previous trial. Black arcs between tissue and disease/biomarker nodes represent tissue-disease associations. Black arcs between protein and tissue nodes represent overexpression of a protein in the tissue. Proteins included in figure are those present in the most enriched pathway for all proteins included in a community. Enrichment was calculated by comparing the proportion of proteins in a community that are members of a pathway to the proportion of proteins outside the community that are members of the same pathway. The Wald statistic was used to find pathways with statistically significant proportion differences. Underlying data for this graph are in table S13.

Next, we created a knowledge graph, incorporating external data to help orient the MR results and augment the results to identify protein-disease patterns. We linked the available MR results to information on tissue expression, drug indications, tissue-trait associations, and pathway enrichment data using Reactome, identifying protein communities belonging to seven distinct biological processes (Figs. 6 and 7 and table S13). We identified an “Antimicrobial peptides” community consisting of REG3A, PGLYRP2, and BPI. The proteins in this pathway were linked to heart failure (HF), dilated cardiomyopathy (DCM), nonischemic cardiomyopathy, and glucose levels (Fig. 7A). The “Viral infection pathways” protein community, including IL-6R, IL-17RA, and SYK among others, was linked to the risk of inflammatory conditions including multiple sclerosis, RA, and SLE, as well as the disease biomarkers, estimated globular filtration rate, and peak expiratory flow (Fig. 7B). We further observed an “Interleukin receptor SHC signaling” community that linked IL-2RA and CSF2RB to risk of various cancers including breast cancer, prostate cancer, and lung cancer, as well as the neurological diseases ALS and Alzheimer’s disease (Fig. 7C). The community enriched for “Ficolin binding” proteins including MASP1 and FCN1 was associated with blood pressure as well as CHD, T2DM, and gout (Fig. 7D). Figure S3 illustrates the other networks identified using this approach.

### Replication and colocalization

Replication data from independent plasma proteomic data were available for 567 of the 1213 multiplicity-corrected MR associations (table S14). Among these, 411 (72%) achieved nominal significance (*P* < 0.05) and showed effect estimates concordant in direction with the original analysis. With a Bonferroni-corrected *P* value of 6.85 × 10^–4^, accounting for the number of proteins available for replication (73), 326 associations (57%) achieved statistical significance with directional concordance. Integrating results across MR, colocalization (table S15), and replication analyses identified a set of 33 associations with consistent support (table S16). These included associations affecting three traits or more: apolipoprotein E (APOE) affecting Lewy body dementia, high-density lipoprotein cholesterol (HDL-C), and T2DM; CD163 affecting low-density lipoprotein cholesterol (LDL-C), HDL-C, and Graves’ disease; SERPING1 (C1 inhibitor) affecting blood pressure, estimated glomerular filtration rate, VTE, and pneumonia; and IL-6R associating with stroke, AF, abdominal aortic aneurysm, blood pressure, hemoglobin A1c (HbA1c), CHD, and pneumonia (table S16).

### Positive controls

From the ChEMBL and TrialTrove data, there were a total of 55 possible positive control protein-disease pairs that have been targeted by compounds at trial phases III or IV (table S17). Of the 55 possible controls, 9 MR associations in our analysis had multiplicity corrected significance, of which 6 (67%) had directional concordance between MR result and trialed drug mechanism. A further 24 MR associations had a *P* < 0.05, of which 16 (67%) had directional concordance with the drug mechanism. MR was thus able to identify 33 (60%) of the positive controls, 22 of which (67%) had directional concordant effect compared to the drug mechanism. Among these, three distinct drugs had received market approval (belimumab, anakinra, and tocilizumab), for which the MR results showed complete directional concordance.

## DISCUSSION

In this study, we assessed associations between 151 immune-related proteins and 64 diseases and disease biomarkers, aiming to prioritize proteins and pathways relevant to multimorbidity. We observed that, on average, immune-related proteins associated with eight diseases or biomarkers (Q1, 4; and Q3, 13), confirming their wide-ranging effects. We identified seven biological pathways, linked to the risk of specific disease groups relevant for multimorbidity, including ficolin binding proteins affecting inflammatory conditions such as atopic dermatitis, pneumonia, and gout, as well as CHD and blood pressure, and the antimicrobial peptide pathway affecting in cardiac traits such as HF and DCM. Our analysis identified 40 druggable but not yet trialed targets relevant for de novo drug development, as well as 73 targets that have been trialed at human clinical phases, as potential candidates for drug repurposing. We furthermore identified 101 drugged or druggable targets affecting multiple disease domains (median, 8; Q1, 4; and Q3, 13), particularly relevant for management of multimorbidity, supporting the development of drugs to treat or prevent multiple related diseases at the same time. Our analyses demonstrated strong validity of the MR findings, supported by consistent results across multiple analytical approaches. More than 70% of associations were replicated in independent proteomic datasets, with additional colocalization support for 33 associations. Additionally, several biologically plausible associations, such as those involving IL-6R and IL-2RA, were among the 60% of possible disease-target positive controls that we successfully reidentified.

Our analysis identified 18 proteins with either predominantly harmful or predominantly protective effects across multiple disease domains. Druggable proteins with a consistent risk increasing direction would require a drug with an inhibitory therapeutic mechanism; this is informative as inhibitors are typically easier to develop than activators. Among these were the druggable proteins CCL17 and CSF1. The chemokine CCL17, which had associations across multiple disease groups including cancers, cardiac outcomes, neurological diseases, and renal outcomes, has been found to be elevated levels in a range of autoimmune and inflammatory diseases (*20*), atopic dermatitis, and cancers, associations that were rediscovered in our study. We further observed that higher values of CCL17 increased the risk of cardiac outcomes including CHD and cardioembolic stroke, which is consistent with a recent mouse model findings identifying CCL17 as a therapeutic target for vascular aging (*21*). The cytokine CSF1 has known associations with cancers, including breast, ovarian, and prostate cancers, with preclinical models suggesting that CSF1 is an important factor in modulating tumor-associated macrophages (*22*–*24*). We now show that the inhibition of CSF1 may additionally reduce inflammation associated with not only other IMI diseases but also neurological diseases such as Parkinson’s disease and ALS.

Sixty-seven percent of the positive control proteins with significant MR associations demonstrated directional concordance with trial effects. Recent studies in transcriptome-wide association studies, a related GWAS-based framework conceptually similar to MR, have shown that “effect flipping” is not unexpected in GWAS-based analyses (*25*). Discordant directions may reflect differences in instrument selection, scaling, population structure, or context-specific biology rather than absence of true relevance. Therefore, incomplete concordance among positive controls does not invalidate the approach, with the majority of positive controls showing concordant directions, therefore providing reasonable validation. At the same time, the subset of proteins showing consistent MR effect directions across diseases remains informative as directional consistency across independent outcomes provides a more stringent signal of broadly aligned effects.

The consistently protective proteins would require a drug with an activator mechanism. Drugs with this mechanism tend to be harder to achieve in drug development, but alternative therapeutic approaches may be possible for proteins with beneficial effects, for example, blockage of an endogenous antagonist or repressor, if present. Proteins with a consistent risk decreasing direction included TLR1, DHX58, and IL-2RA. Deficiencies in TLR1 are linked to a depleted immune response, and small-molecule TLR1 agonists are in late-stage trials for treatment of cancers such as melanomas and infectious diseases (*26*, *27*). Our study showed that increased TLR1 values decreased the risk of cervical cancer, in line with the trial mechanism and increased the risk of non-Hodgkin’s lymphoma, the opposite of the trial mechanism. IL-2RA has been targeted by daclizumab as a potential treatment for multiple sclerosis, blocking the receptor and downstream immune activation. However, the drug was withdrawn as many patients developed serious inflammatory brain disorders including inflammatory encephalitis and meningoencephalitis (*28*, *29*). Our study shows increased levels of IL-2RA being protective against most diseases or biomarkers, such as HF, DCM, as well as Alzheimer’s disease. While the diseases are distinct from the trial adverse events, it is interesting to note the concordance of risk direction between the MR results and the trial adverse events. In addition, HF, DCM, and Alzheimer’s disease are all suspected of having an inflammatory component (*30*–*32*). Low-dose IL-2 therapy is now emerging as a therapy to treat various inflammatory diseases including ischemic heart disease and ulcerative colitis (*33*). The observation that proteins in the constant direction set are enriched for pathways involved in TLR signaling and MHC class I antigen presentation is potentially interesting. TLRs are involved in innate immune processes, and their up-regulation can enhance MHC class I antigen presentation and cross-presentation of exogenous antigens on MHC class I molecules. Dysregulation in these pathways would lead to auto-inflammatory processes or cross-presentation of self-antigens and autoimmunity.

The network analysis suggests that protein-disease associations should not always be considered in isolation and that multiple proteins involved in pathways could be important in understanding the risk of disease onset and progression. The antimicrobial peptide (AMP) community, characterized by proteins including REG3A, PGLYRP2, BPI, and PI3. AMPs, traditionally regarded as antibiotics, are now known to modulate immune and inflammatory responses more broadly. Their grouping in our network analyses, affecting HF, nonischemic CM, and DCM, supports emerging evidence for mechanisms linking infection, inflammation, and cardiac dysfunction, presenting them as potential therapeutic targets for inflammation-driven cardiovascular disease (*34*). The ficolin binding community linked FCN1 and MASP1 to inflammatory conditions such as atopic dermatitis, pneumonia, and gout, as well as CHD and blood pressure. Ficolins are soluble innate immune recognition proteins that are involved in the triggering of the lectin arm of the complement system, thereby modulating downstream inflammatory responses. The fact that ficolin-associated proteins cluster with disease across various groups suggests lectin-pathway activation as a shared inflammatory mechanism across these conditions and exemplifies the interconnectedness of inflammatory pathways in multimorbidity.

Our study identified 113 drugged or druggable immune-related proteins: 40 druggable but not yet drugged proteins as potential de novo targets, 73 targets that have been targeted in human trials and present opportunities for drug repurposing, and, among these, 101 druggable proteins that have effects across multiple disease groups. For example, FGCR2B, for which our study found associations with the IMID SLE (risk increasing) and RA (risk decreasing), is undergoing phase 2/3 trials against obexelimab for treatment of various outcomes including anemia, immunoglobulin G4–related diseases, and SLE (*35*–*37*). Our study found that it has effects in not only these IMIDs but also across other disease groups, with significant associations with cardioembolic stroke, atrial fibrosis, prostate cancer, and Parkinson’s disease. Similarly, IL-17RD, the protein with the most associations in our study, was associated with both RA and SLE in the IMI disease group, with both AF and T2DM in the cardiometabolic disease group, as well as with prostate cancer and Parkinson’s disease. IL-17A, for which IL-17RD is a ligand, is the target for secukinumab (*38*), a licensed drug for the treatment of psoriasis, and our study suggests that its widespread effects across disease groups could make it a key candidate for drug repurposing opportunities. CXCL11, which had concordantly harmful effects across diseases and biomarkers, has been previously associated with tumor suppression in patients with cancer. Our study supports these findings, associating CXCL11 not only with multiple cancers including prostate, lung, and endometrial cancer but also with diseases across various groups including stroke, CHD, and RA. This presents CXCL11 as a target for de novo drug development, not only in cancers but also potentially across other disease groups. Additionally, our study rediscovered some significant protein-trait associations that have been trialed in stage III clinical trials. Associations between IL-6Rs were significantly associated with diseases already under trial using IL-6R inhibition, including HF and CHD. The association between IL-2RA and SLE was also trialed with the drug ustekinumab, and CSF1 was also part of a phase 3 trial for the treatment of ALS.

The protein-disease associations with an overlap of MR, colocalization, and replication analyses have the strongest level of genetic evidence, suggesting that they may represent high-priority candidate targets for further investigation. Notably, the association between APOE and dementia aligns with existing evidence (*39*), highlighting how APOE interacts with lipoprotein receptors to regulate neuronal lipid metabolism and energy use, linking lipid dysregulation with neurodegeneration. Similarly, IL-6R, the licensed target of tocilizumab for RA, showed robust evidence across a range of cardiometabolic and inflammatory outcomes, including AF, CHD, and pneumonia, indicating potential opportunities for therapeutic repurposing. SERPING1, IL-2RA, and TYRO3 showed strong and directionally consistent associations, including associations with AF, HF, Lewy body dementia, and breast cancer, highlighting them as promising candidates for further translational investigation.

There are some potential limitations to our study that require consideration. First, our analyses were limited by the coverage of the considered proteomics GWAS. As a result, some key proteins involved in inflammation and immunity, for example, TNF-α that is a licensed drug target for RA, were not present. Similarly, well-established inflammatory diseases, including inflammatory bowel disease, ulcerative colitis, and Crohn’s disease, were discounted because of an insufficient number of variants matching the proteomics GWAS. In addition, although some of the proteins in our study may be implicated in infectious diseases such as tuberculosis, where inflammatory pathways play a central role in host response, infectious outcomes were not comprehensively evaluated here. This was due to both our primary focus on chronic, noncommunicable conditions and the limited availability of well-powered GWAS with robust genome-wide significant signals for these traits. As such, our current analysis provides a strong basis for further exploration in terms of both considered proteins and considered traits. Another potential limitation is that the defined disease groups reflect different levels of granularity where, for example, the group “stroke” (consisting of large artery stroke, ischemic stroke, any stroke, cardioembolic stroke, small vessel stroke, and stroke volume) has a much narrower definition than the group “autoimmune diseases” (consisting of eight outcomes including RA, SLE, and multiple sclerosis). While this partly reflects the available GWAS data, different categorizations are possible, which, in turn, may lead to distinct inference about disease pleiotropy. For example, broader disease categories could capture more heterogeneous biological mechanisms and overlapping pathways than in narrower categories. Furthermore, some outcomes within and across disease groups represent closely related or partially overlapping phenotypes (e.g., HF/DCM), which may reflect different clinical manifestations or measurements of the same underlying disease process. As a result, in cases such as these, associations observed across multiple related outcomes should be interpreted as evidence of consistency across a disease spectrum rather than as effects on distinct diseases. Next, while tissue-specific data were included in the knowledge graph, the protein-quantitative trait locus (pQTL) studies used in the study only measure protein levels in the plasma, with no tissue-specific measurements. This may result in an inaccurate model of direct effects of protein modulation, and we may see some effect direction flipping due to leakage into the plasma reflecting pathology in the periphery (*16*). Additionally, in our analysis of proteins with consistently harmful or beneficial effects, we purposefully combined results supported to varying degrees (for example, by replication, colocalization, or relevant tissue expression). This approach ensures that potential future experimental studies are informed about the full range of possible consequences of protein perturbation and can assess these comprehensively to confirm or refute relationships with disease risk.

Last, some caution should generally be applied when translating genetic findings into drug interventions. For example, MR is often interpreted in terms of lifelong genetic perturbation, whereas therapeutic interventions are often prescribed later in life and potentially for a limited period. Additionally, some of the key findings in our study involved inflammatory cytokines such as IL-2RA and TNF. The effects of cytokines are involved in multiple pleiotropic pathways, where circulating levels are susceptible to stimuli such as metabolic stress or infection, meaning that identified associations may reflect systemic immune activation rather than disease-specific mechanisms. In contrast, regulators such as TYRO3 and SERPING1 may mark more disease-specific pathways. Distinguishing between these two scenarios, for example, by conducting tissue-specific or in vivo analyses is therefore essential to understand the therapeutic implications of translating MR findings. This does not necessarily imply that findings from inflammatory cytokines should be dismissed but rather highlights the (general) need for confirmatory preclinical validation of target involvement in disease causing mechanisms. A final consideration is that the proteomics GWAS used in the current study used in the current study did not all use the same arrays (see table S18), each not only with distinct protein coverage but also with different levels of measurement accuracy of potential for assay artifacts. For the subset of proteins available in multiple GWAS, we explored replication. Nevertheless, we wish to caution readers that due to these differences in assays, direct comparisons in terms of effect magnitudes are difficult. As discussed by Schmidt *et al*. (*16*) and (*15*), findings from human genetics on protein-disease associations are not anticipated to directly reflect effect sizes of protein perturbation through pharmacological intervention but rather provide important information on required drug mechanism of action (inhibiting or activating) through consideration of effect direction. Regardless of the presented limitations, we anticipate that our results provide a strong basis to prioritize and further validate the identified druggable leads.

In conclusion, we found that immune-related proteins are strongly linked to the development of multimorbidity, which provides actionable opportunities for de novo drug development for multiple diseases together as well as for indication expansion and drug repurposing. To accelerate this, we have identified 113 druggable proteins with 73 proteins relevant for drug repurposing, including 101 proteins affecting multiple disease groups. Among these, we identified a subset of 33 protein-disease pairs with strong genetic evidence of causality.

## MATERIALS AND METHODS

### Selecting genetic instruments for immune proteins

Data for plasma proteins were taken from eight GWAS that identified pQTLs, which provided genetic association summary statistics (table S18). A single pQTL source per protein was used, and if pQTLs for the same protein were identified in more than one study, then we used summary effect estimates from the study with the largest sample size (table S19).

To restrict the analysis to immune-related proteins, we examined the intersection of all proteins with valid genetic instruments for MR against the UniProt database (*40*), retaining proteins matching any of the UniProt keyword terms “cytokine,” “interleukin,” “inflammasome,” “inflammatory response,” or “innate immunity.” We cross validated these proteins against Gene Ontology (GO) terms “cytokine receptor,” “innate immune,” and “interleukin receptor activity.” This approach identified 151 proteins for further analysis (see table S19 for proteins, studies from which they were taken, and corresponding sample sizes).

### Selecting disease biomarkers and outcomes

Clinical experts (A.D.H. and F.W.A.) prioritized disease outcomes for inclusion based on existing literature on multimorbidity and experience in health care settings (*2*). These outcomes included both disease diagnoses and biomarkers deemed relevant for either multimorbidity or inflammation and innate immunity. The data for the resulting 50 disease outcomes and 14 biomarkers were sourced from independent GWAS (table S19). The diseases outcomes came from disease groups including IMIDs (e.g., SLE and RA), cardiac diseases (e.g., AF and HF), and cancers (e.g., breast cancer and prostate cancer), among others, and the biomarkers included, for example, triglyceride levels, HDL-C, and HbA1c concentrations. Certain infectious diseases (e.g., pneumonia) were also included as their risk could be increased by drugs with an anti-inflammatory effect.

### MR analysis

Cis-MR analyses were conducted by identifying variants located in or around the protein-encoding gene using a 200–kilo–base pair (kbp) window to allow a balance between including sufficient cis-acting variants and preventing influence by variant from neighboring genes (*41*) and an *F* statistic of 15. Variants with a minor allele frequency (MAF) below 1% were removed to reduce the risk of inflated effect estimates. Palindromic single-nucleotide polymorphisms, with MAF near 0.50, where identification of the effect allele can be ambiguous, were excluded (*42*). The remaining variants were clumped to a pairwise coefficient of determination (*R*^2^) of 0.40. A random sample of 5000 UK Biobank (UKB) participants with British ancestry (*43*) was used as reference, with the subset size allowing for stable LD estimates for variants with MAF of 0.01 or larger.

MR analysis was used to estimate the association of each protein evaluated on each of the 64 selected diseases and biomarkers. Two MR estimators were used: the inverse variance weighted method (IVW) (*44*) and MR-Egger (MRE) that corrects for horizontal pleiotropy (*45*). Both estimators were implemented using a generalized least squares solution, accounting for residual linkage disequilibrium using the UKB as reference data. The Rücker model selection framework (*46*), which uses a goodness-of-fit statistic to compare models, was used to select the estimate (IVW or MRE) most supported by the available data. The potential influence of pretranslational horizontal pleiotropy bias (which arises when the genetic instrument is associated with disease independent of the protein of interest) was further limited by excluding variants with a leverage statistic larger than three times the mean leverage and by excluding variants with an outlier (i.e., chi-square) statistic larger than 11 (*47*). To reduce the risk of reverse causality, we applied Steiger filtering excluding variants more strongly associated with outcomes than the considered protein. Last, to ensure that we had sufficient data to accurately model the protein effects, we discounted analyses with few than five genetic variants.

We present MR results as mean differences for continuous traits (i.e., biomarkers) or as odds ratios for binary outcomes (i.e., diseases). Effect estimates are presented with 95% confidence intervals and *P* values. Results were evaluated for significance against a Bonferroni corrected *P* value threshold of 5.16 × 10^−6^, based on the product of the number of considered proteins and traits (151 × 64 = 9664).

### Annotating identified associations

Each outcome was classified into one of 13 disease groups (table S21). For example, RA and Graves’ disease were grouped as “autoimmune.” For biomarkers, we additionally recorded the anticipated “beneficial direction,” indicating whether higher or lower values of the protein of interest were likely to increase or disease risk; for example, a decreasing effect on LDL-C was indicated as being beneficial (table S21). For all disease outcomes, an increase in risk was marked as “harmful.”

The Human Protein Atlas (version 20) (*48*, *49*) was used to extract information on tissue expression and specificity for each of the analyzed proteins, identifying tissues, in which they had larger than average expression (Supplementary Method 1).

Tissues were linked to diseases and biomarkers using the method by Lage *et al.* (*50*) (Supplementary Method 2). Briefly, this method measures the co-occurrence of the tissue and trait keywords in abstracts in PubMed (*51*), resulting in a score of 0 or larger. Synonyms for tissues were identified using the BRENDA tissue ontology (*52*), and, for traits, synonyms were extracted from the Unified Medical Language System metathesaurus (version 2022AB) (*53*).

Proteins were classified as “drugged” if they had been targeted by a drug at any human clinical trial phase and “druggable” if included in a previously published set of druggable proteins, but not yet trialed as drug targets (*54*). Existing drug trials and clinical indications were identified on the basis of data from ChEMBL (*55*). Additionally, TrialTrove (*56*), a large, manually curated database for clinical trials, was queried to extract information on ongoing or completed drug trials for each protein. Receptors for each circulating protein (and ligands for each soluble form of a receptor protein) were extracted using the Search Tool for Retrieval of Interacting Genes/Proteins (STRING) database of protein-protein interactions (table S22) (*57*), and trials or known indications were linked to a protein if they had been reported for any of its interactors.

We mapped each protein to one or more biological pathways using information from Reactome. The pathway data from Reactome (*58*) represent manually curated, peer-reviewed evidence from human experiments, which include cross-references to information in the GO database (*59*).

### Identifying positive controls

Potential positive controls were identified by cross-referencing protein-disease pairs evaluated in the MR analysis with late-stage clinical trial data. For each protein-disease pair with an MR estimate, we searched for phase 3 or 4 trials targeting the protein itself or its known biological interactors (defined using the STRING database) for the same disease indication. Trial data were obtained from ChEMBL and TrialTrove and further manually screened by clinical experts. For protein-disease pairs with identified late-stage trials, we evaluated the statistical significance of the corresponding MR estimate (both nominal *P* < 0.05 and after multiple-testing correction).

Directional concordance was defined as agreement between the drug’s mechanism of action and the direction of the MR estimate. Specifically, if increased protein levels were associated with higher disease risk, the concordance required trialed compounds to act as inhibitors; conversely, if increased protein levels were protective, then concordance required trialed compounds to act as agonists.

### Prioritization of proteins with shared pleiotropic disease/biomarker effects

We prioritized the immune proteins based on a univariable analysis on the MR results of protein values significantly associating with disease and biomarkers. We first determined the number of times each immune-related protein was associated with a disease or biomarker. We then took each disease, in turn, identified the proteins associated with this disease, whether each protein was drugged or druggable (see above and table S3), and whether the protein was associated with each of the disease groups (table S21). Using the “beneficial direction” annotations previously assigned to each disease/biomarker (table S21), we additionally identified proteins with a predominantly harmful or predominantly beneficial effect (using an arbitrary cutoff of 75% directional concordance).

To illustrate the pleiotropic effects of immune-related plasma proteins, we next considered proteins affecting an indexing outcome and recorded their pleiotropic effects on other multimorbidity-related diseases and disease biomarkers. First, we considered immune proteins associated with SLE, RA, and PBC as a group of known IMIDs. We next explored the influence of immune-related proteins on the occurrence of the cardiometabolic diseases AF, CHD, and T2DM, for which low-grade inflammation has been implicated in their pathogenesis. Last, this was expanded to consider outcomes with less well-established inflammatory involvement such as neurological diseases (e.g., Alzheimer’s disease and Parkinson’s disease) and cancers including lung, prostate, and breast cancer and breast cancer.

### Protein network analytics

We created a graph database using Neo4j version 4.4.8 (*60*), where proteins (identified by UniProt entry names), traits [identified by medical subject headings (MeSH) terms; (*61*)], and tissues were included as nodes and where edges were used to represent relationships. Specifically, tissue-trait relationships were included if the association score, calculated using the previously described method by Lage *et al.*, was above 8; protein-tissue arcs were included for overexpressed proteins (i.e., tissues with a significantly higher expression for a protein than the average expression of that protein); edges between the protein and outcome were included for the MR associations that passed multiplicity correction. Known drug effects were modeled using arcs between the protein drug target and the indicated disease. To facilitate the construction of the graph, tables S11 and S12 provide the comprehensive list of all nodes and their corresponding edges, respectively.

Neo4j graph data science library (version 2.1) (*60*) was used to identify graph communities of proteins and disease/biomarkers. Specifically, the Louvain algorithm (*62*) was used, which aims to maximize the modularity of a community. Modularity represents the density of the connections in a graph and is high when the relationships within communities are denser than those between communities and, as such, naturally identifies clusters of proteins and disease/biomarkers. The communities were subsequently annotated using Reactome pathway data (version 89). Enriched pathways were identified by comparing the pathway membership of the proteins within the community against those outside the community. Enrichment was formally evaluated by testing for the difference in proportions using a *P* value threshold of 0.05 (*47*). For each community, the pathway with the highest proportion difference of all enriched pathways, where at least two proteins in the pathway were present in the community, was extracted. In the case where the pathway with highest enrichment covered too broad a biological process to be informative (e.g., “disease”), the pathway with the second highest enrichment was extracted if available.

### Replication analysis

To strengthen support for our MR findings, we conducted replication analysis for proteins measured in more than one of the eight pQTL studies included in our primary analyses. For each protein, we selected the study with the largest sample size for the main analysis and used estimates from the other studies for replication. We additionally applied the previously described cis*-*MR techniques to the UKB plasma pQTL (*63*) (*n* = 54,219) and used these associations as an additional source of replication evidence derived from a different protein assay technique. Associations identified in the primary MR analyses were considered replicated if we observed a directionally consistent association and the *P* value was smaller than 0.05. We additionally considered a more conservative *P* value of 6.85 × 10^−4^, accounting for the 73 proteins for which replication data were available.

### Colocalization

To further assess whether the genetic associations for the protein and outcome were driven by the same underlying causal variant, we conducted colocalization analyses for each available protein-outcome pair, using the same data as used in the primary MR analyses, supplemented with the UKB pQTL data. We included variants from a 500-kbp flanking window of the protein-encoding gene, but, as all variants at a locus should be considered as the potential causal variant, following coloc guidance (*64*, *65*), we did not filter for MAF or minimum *P* value. We considered variants to be colocalized, with a shared causal variant, if the posterior probability was 0.8 or larger.

## Supplementary Materials

### The PDF file includes:

Supplementary Methods 1 and 2 Figs. S1 to S3 Legends for tables S1 to S22 References
