# Supplementary material for: The immunoproteome and multimorbidity: A Mendelian randomization study
Source: Sci Adv. 2026 May 20;12(21):eadz7117. doi: 10.1126/sciadv.adz7117 (PMC13189124; doi:10.1126/sciadv.adz7117)
Supplement: Supplementary file 1 — Supplementary Methods 1 and 2 Figs. S1 to S3 Legends for tables S1 to S22 References [file sciadv.adz7117_sm.pdf]

Science Advances
AAAS

# Supplementary Materials for

## The immunoproteome and multimorbidity: A Mendelian randomization study

Nikita Hukerikar *et al.*

Corresponding author: Nikita Hukerikar, nikita.hukerikar.21@ucl.ac.uk

*Sci. Adv.* **12**, eadz7117 (2026)
DOI: 10.1126/sciadv.adz7117

**The PDF file includes:**

Supplementary Methods 1 and 2
Figs. S1 to S3
Legends for tables S1 to S22
References

**Other Supplementary Material for this manuscript includes the following:**

Tables S1 to S22

# Supplementary Materials

Method 1: Method for quantifying specificity of gene expression.

To quantify the specificity of expression of a gene in a given tissue, compared to other tissues, z-scores were calculated for individual gene-tissues pairs(*74*) using the following formula:

$$z_i = \frac{x_i - \mu}{\sigma}, \mu = \frac{\sum_{i=1}^{n} x_i}{n}, \sigma = \sqrt{\frac{\sum_{i=1}^{n}(x_i - \mu)^2}{n-1}}$$

Where $x_i$ is the gene expression in the $i^{th}$ tissue, $\mu$ is mean expression value of the gene across all tissues, $\sigma$ is the standard deviation, and $n$ is the number of tissues.

Method 2: Method for calculating tissue-trait associations using PubMed abstracts.

This method, adapted from the method proposed by Lage et al (*50*), was used to measure the literature co-occurrence between an outcome and a tissue. First, the PubMed abstracts dataset (available at https://ftp.ncbi.nlm.nih.gov/pubmed/baseline/), which contains abstracts and keywords for all PubMed articles was downloaded and loaded into a database. For any given tissue-outcome pair, the database was queried for the number of abstracts mentioning both the outcome and the tissue, or any synonyms for both. To obtain synonyms for the tissues, the BRENDA tissue ontology(*52*) was used, and for the outcomes, synonyms were obtained from the UMLS metathesaurus(*53*).

Having obtained the abstract co-occurrence counts for each term, and for each outcome-tissue pair $(X_{i,}Y_j)$, pairwise Ochiai's coefficient $(O_c)$, a measure of similarity, was calculated. Subsequently a final maximal association score (MAS) was calculated by normalising the $O_c$ score per adverse effect. See formula below.

$$O_c(X_{i,}Y_j) = \sqrt{\frac{C(X_{i,}Y_j)^2}{C(X_i) \cdot C(Y_j)}} \, , MAS(X_{i,}Y_j) = 100\frac{O_c(X_{i,}Y_j)}{\sum_p^n O_c(X_{i,}Y_p)}$$

Where $C(X_i)$ is the number of abstracts mentioning the outcome $X_i$, $C(Y_j)$ is the number of abstracts mentioning the tissue $Y_j$, $C(X_{i,}Y_j)$ is the number of abstracts mentioning both terms and *n* is the total number of tissues.

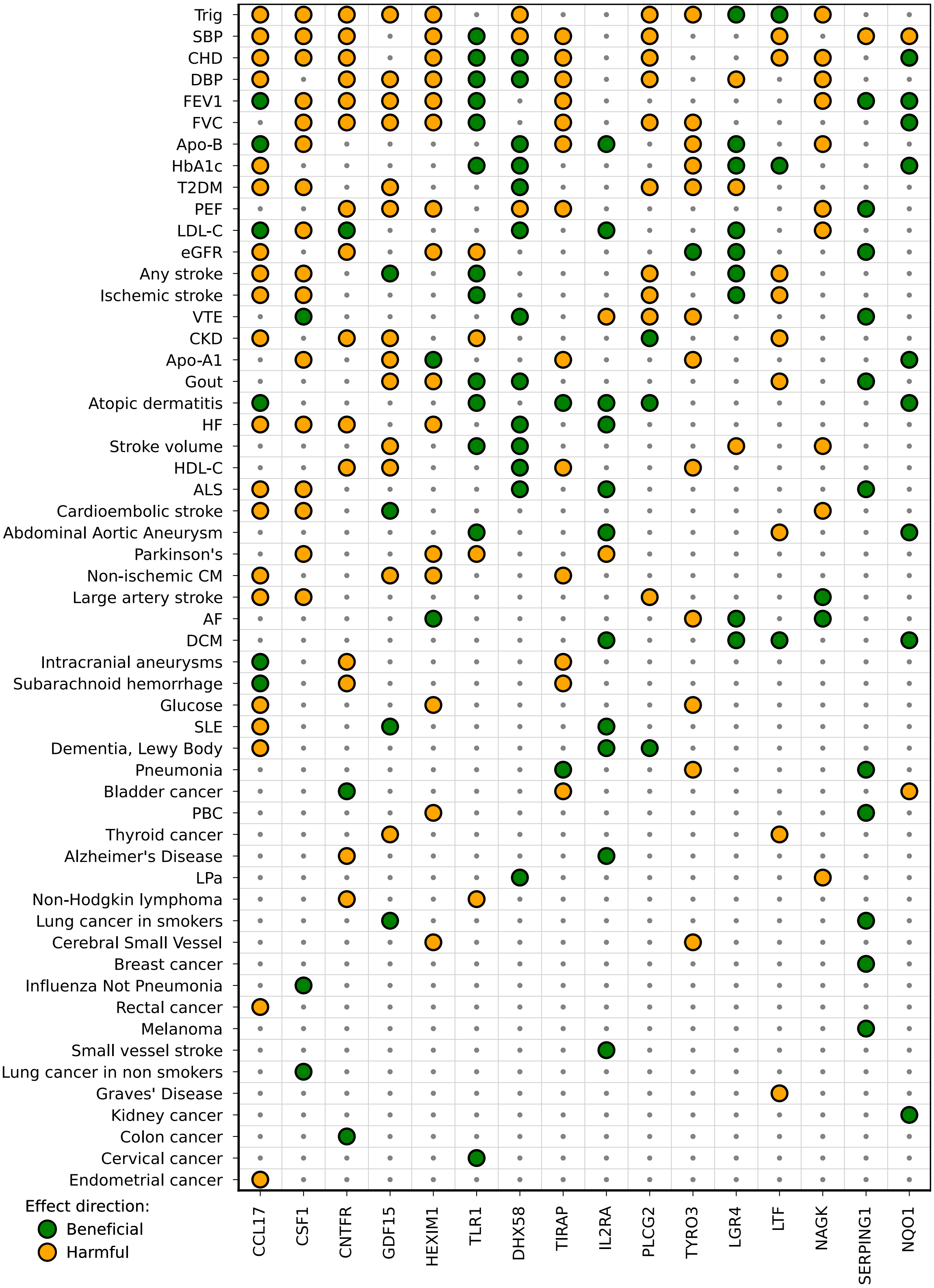


**Supplementary Figure 1:** Individual associations for proteins with concordant effect directions (75% or more of significant associations either harmful or beneficial). Results were evaluated for significance against a Bonferroni corrected p-value threshold of 5.16×10-6

based on the number of proteins and outcomes (Table S1). Effect estimates are reported as odds ratios. The effect estimates are based on *cis*-Mendelian randomisation (MR) analysis with a p-value smaller than a multiplicity corrected threshold of $5.16\times10^{-6.}$ The MR analyses sourced GWAS on plasma protein value from Said *et al.*(*66*)(n: 575,531), Ferkingstad *et al.*(*67*) (n: 35,559), Gilly *et al.*(*68*) (n: 1,328), Sun *et al.* (n: 3,301)(*69*), Folkersen *et al.* (n: 30,931)(*70*), Yang *et al.* (n: 835)(*71*), Yao *et al.* (n: 6861)(*72*)*,* Ahola-Olli *et al.* (n:8293). For the source GWAS on disease and disease-biomarkers please refer to Table S20. The numerical data underlying this illustration are available in Table S4.

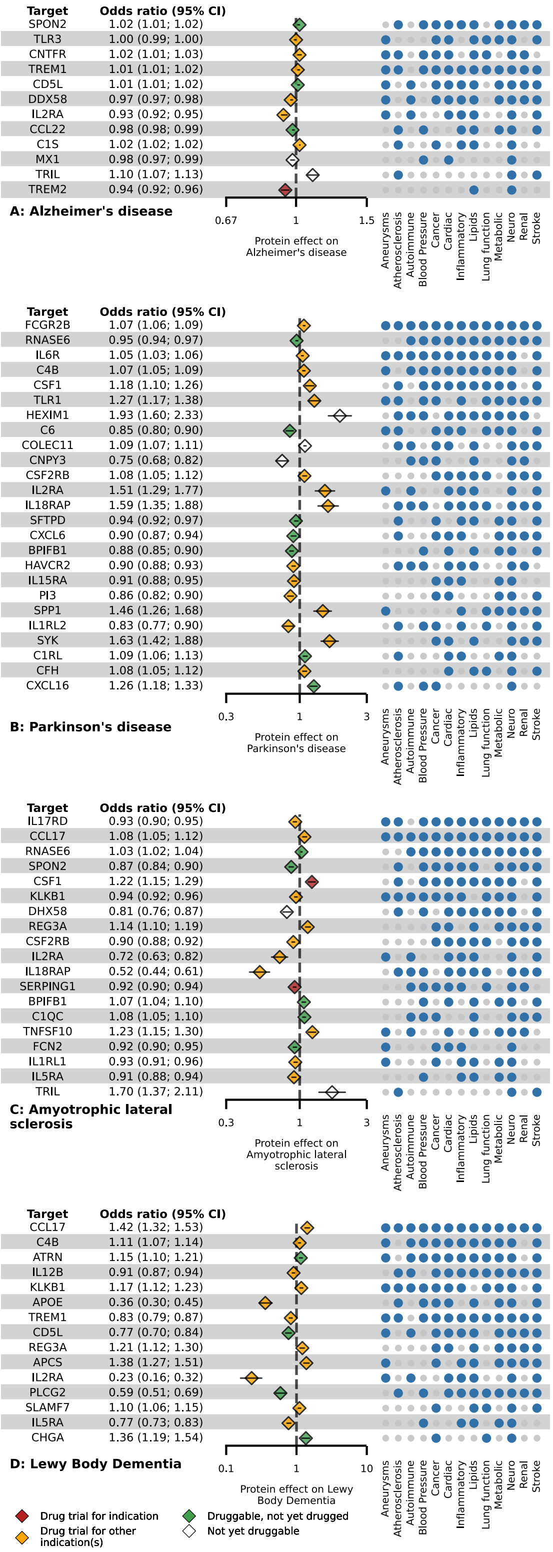


**Supplementary Figure 2** Mendelian randomisation estimates for neurological diseases

N.b. The effect estimates are based on *cis*-Mendelian randomisation (MR) analysis with a p-value smaller than a multiplicity corrected threshold of $5.16\times10^{-6}$. The MR analyses sourced GWAS on plasma protein value from Said *et al.*(*66*)(n: 575,531), Ferkingstad *et al.*(*67*) (n: 35,559), Gilly *et al.*(*68*) (n: 1,328), Sun *et al.* (n: 3,301)(*69*), Folkersen *et al.* (n: 30,931)(*70*),

Yang *et al.* (n: 835)(*71*), Yao *et al.* (n: 6861)(*72*)*,* Ahola-Olli *et al.* (n:8293)(*73*). For the source GWAS on disease and disease-biomarkers please refer to Table S20. The numerical data underlying this illustration are available in Table S9. The effect direction is based on a unit increase in protein concentration. Incidence matrix indicates other disease groups in which at least one outcome is associated with a protein. Marker colours on plot indicated the drug trial status of the protein as a target as per TrialTrove and ChEMBL. Sub-figures A, B, C and D show associations for Alzheimer's disease, Parkinson's disease, amyotrophic lateral sclerosis and Lewy body dementia respectively.

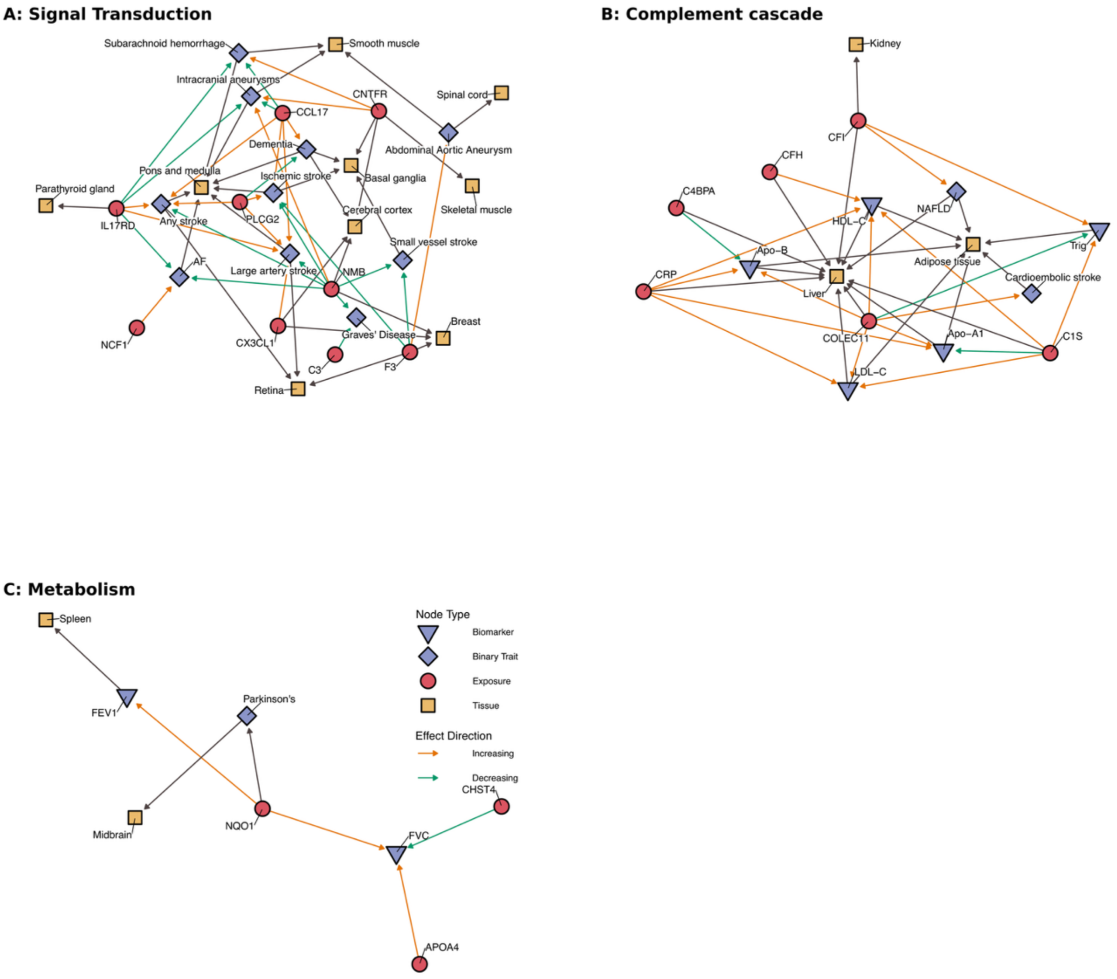


**Supplementary Figure 3** Proteins in enriched pathways for graph communities

n.b. Orange and green arcs represent significant Mendelian randomisation (MR) arcs between protein and disease/biomarker nodes. Black arcs between protein and disease/biomarker nodes represent a previous trial. Black arcs between tissue and disease/biomarker nodes represent tissue-disease associations. Black arcs between protein and tissue nodes represent over-expression of a protein in the tissue. Proteins included in figure are those present in the most enriched pathway for all proteins included in a community. Enrichment was calculated by comparing the proportion of proteins in a community that are members of a pathway to the proportion of proteins outside the community that are members of the same pathway. The Wald statistic was used to find pathways with statistically significant proportion differences ($p<0.05$). Underlying data can be found in Table S13.

**Supplementary Tables**

Table S1: **All significant MR associations**. Results were evaluated for significance against a Bonferroni corrected p-value threshold of 5.16×10-6 based on the number of proteins and outcomes. Effect estimates are reported as odds ratios. Analysis model used is either inverse variance weighted (IVW) or MR-Egger, as selected by the Rücker model selection framework. Abbreviations: AF (Atrial Fibrillation), ALS (Amyotrophic lateral sclerosis), AMD (Age-related macular degeneration), Apo-A1 (Apolipoprotein A1), Apo-B (Apolipoprotein B), CHD (Coronary heart disease), CKD (Chronic kidney disease), DBP (Diastolic blood pressure), DCM (Dilated cardiomyopathy), FEV1 (Forced expiratory volume), FVC (Forced vital capacity), HDL-C (High-density lipoprotein cholesterol), HF (Heart failure), HbA1c (Haemoglobin A1c), LDL-C (Low-density lipoprotein cholesterol), LPa (Lipoprotein (a)), NAFLD (Non-alcoholic fatty liver disease), Non-ischemic CM (Non-ischemic cardiomyopathy), PBC (Primary biliary cirrhosis), PEF (Peak expiratory flow), SBP (Systolic blood pressure),SLE (Systemic lupus erythematosus), T2DM (Type 2 diabetes mellitus), Trig (Triglycerides), VTE (Venous thromboembolism), eGFR (Estimated glomerular filtration rate).

Table S2: **Top 10 exposures with highest number of significant MR associations**. Results were evaluated for significance against a Bonferroni corrected p-value threshold of 5.16×10-6 based on the number of proteins and outcomes (Table S1). The number of associations with diseases from each of the 13 disease groups (Table S19) are also included.

Table S3: **All exposures with druggability status.** Druggability status is as per ChEMBL and TrialTrove, number of indications as per ChEMBL, and max trial phase as per TrialTrove.

Table S4: **All significant MR associations for proteins with concordant effect directions** (75% or more of significant associations either harmful or beneficial). Results were evaluated for significance against a Bonferroni corrected p-value threshold of 5.16×10-6 based on the number of proteins and outcomes (Table S1). Effect estimates are reported as odds ratios. Analysis model used is either inverse variance weighted (IVW) or MR-Egger, as selected by the Rücker model selection framework. Abbreviations: AF (Atrial Fibrillation), ALS (Amyotrophic lateral sclerosis), AMD (Age-related macular degeneration), Apo-A1 (Apolipoprotein A1), Apo-B (Apolipoprotein B), CHD (Coronary heart disease), CKD (Chronic kidney disease), DBP (Diastolic blood pressure), DCM (Dilated cardiomyopathy), FEV1 (Forced expiratory volume), FVC (Forced vital capacity), HDL-C (High-density lipoprotein cholesterol), HF (Heart failure), HbA1c (Haemoglobin A1c), LDL-C (Low-density lipoprotein cholesterol), LPa (Lipoprotein (a)), NAFLD (Non-alcoholic fatty liver disease), Non-ischemic

CM (Non-ischemic cardiomyopathy), PBC (Primary biliary cirrhosis), PEF (Peak expiratory flow), SBP (Systolic blood pressure),SLE (Systemic lupus erythematosus), T2DM (Type 2 diabetes mellitus), Trig (Triglycerides), VTE (Venous thromboembolism), eGFR (Estimated glomerular filtration rate).

Table S5: **Enriched pathways for proteins with concordant effect direction**. Enriched pathways identified by comparing the pathway membership of the proteins with concordant effect directions (Table S4) against those without. Enrichment formally evaluated by testing for the difference in proportions using a false-positive rate of 0.05

Table S6: **All significant MR associations for the group of immune mediated inflammatory diseases: systemic lupus erythematosus (SLE), rheumatoid arthritis, primary biliary cholangitis (PBC).** Results were evaluated for significance against a Bonferroni corrected p-value threshold of 5.16×10-6 based on the number of proteins and outcomes (Table S1). Effect estimates are reported as odds ratios. Analysis model used is either inverse variance weighted (IVW) or MR-Egger, as selected by the Rücker model selection framework. Results are annotated with druggability status and number of known indications as per ChEMBL and TrialTrove. Trial data is taken from TrialTrove.

Table S7: **All significant MR associations for the group of cardiometabolic diseases: atrial fibrillation (AF), coronary heart disease (CHD) and type 2 diabetes (T2DM).** Results were evaluated for significance against a Bonferroni corrected p-value threshold of 5.16×10-6 based on the number of proteins and outcomes (Table S1). Effect estimates are reported as odds ratios. Analysis model used is either inverse variance weighted (IVW) or MR-Egger, as selected by the Rücker model selection framework. Results are annotated with druggability status and number of known indications as per ChEMBL and TrialTrove. Trial data is taken from TrialTrove.

Table S8: **All significant MR associations for the group of cancers: lung cancer in smokers, prostate cancer and breast cancer**. Results were evaluated for significance against a Bonferroni corrected p-value threshold of 5.16×10-6 based on the number of proteins and outcomes (Table S1). Effect estimates are reported as odds ratios. Analysis model used is either inverse variance weighted (IVW) or MR-Egger, as selected by the Rücker model selection framework. Results are annotated with druggability status and number of known indications as per ChEMBL and TrialTrove. Trial data is taken from TrialTrove.

Table S9: **All significant MR associations for the group of neurological diseases: Alzheimer's disease, Parkinson's disease, amyotrophic lateral sclerosis (ALS).** Results were evaluated for significance against a Bonferroni corrected p-value threshold of 5.16×10-6 based on the number of proteins and outcomes (Table S1). Effect estimates are reported as odds ratios. Analysis model used is either inverse variance weighted (IVW) or MR-Egger,

as selected by the Rücker model selection framework. Results are annotated with druggability status and number of known indications as per ChEMBL and TrialTrove. Trial data is taken from TrialTrove.

Table S10: **Trials data for all proteins**. Trials data was taken from TrialTrove. Abbreviations: AF (Atrial Fibrillation), ALS (Amyotrophic lateral sclerosis), AMD (Age-related macular degeneration), Apo-A1 (Apolipoprotein A1), Apo-B (Apolipoprotein B), CHD (Coronary heart disease), CKD (Chronic kidney disease), DBP (Diastolic blood pressure), DCM (Dilated cardiomyopathy), FEV1 (Forced expiratory volume), FVC (Forced vital capacity), HDL-C (High-density lipoprotein cholesterol), HF (Heart failure), HbA1c (Haemoglobin A1c), LDL-C (Low-density lipoprotein cholesterol), LPa (Lipoprotein (a)), NAFLD (Non-alcoholic fatty liver disease), Non-ischemic CM (Non-ischemic cardiomyopathy), PBC (Primary biliary cirrhosis), PEF (Peak expiratory flow), SBP (Systolic blood pressure),SLE (Systemic lupus erythematosus), T2DM (Type 2 diabetes mellitus), Trig (Triglycerides), VTE (Venous thromboembolism), eGFR (Estimated glomerular filtration rate)

Table S11: **All nodes, with node types, used to construct the Neo4j knowledge graph used for protein network analytics.** Abbreviations: AF (Atrial Fibrillation), ALS (Amyotrophic lateral sclerosis), AMD (Age-related macular degeneration), Apo-A1 (Apolipoprotein A1), Apo-B (Apolipoprotein B), CHD (Coronary heart disease), CKD (Chronic kidney disease), DBP (Diastolic blood pressure), DCM (Dilated cardiomyopathy), FEV1 (Forced expiratory volume), FVC (Forced vital capacity), HDL-C (High-density lipoprotein cholesterol), HF (Heart failure), HbA1c (Haemoglobin A1c), LDL-C (Low-density lipoprotein cholesterol), LPa (Lipoprotein (a)), NAFLD (Non-alcoholic fatty liver disease), Non-ischemic CM (Non-ischemic cardiomyopathy), PBC (Primary biliary cirrhosis), PEF (Peak expiratory flow), SBP (Systolic blood pressure),SLE (Systemic lupus erythematosus), T2DM (Type 2 diabetes mellitus), Trig (Triglycerides), VTE (Venous thromboembolism), eGFR (Estimated glomerular filtration rate)

Table S12: **All edges, with edge types, used to construct the Neo4j knowledge graph used for protein network analytics.** Edges are defined as source and target nodes. Abbreviations: AF (Atrial Fibrillation), ALS (Amyotrophic lateral sclerosis), AMD (Age-related macular degeneration), Apo-A1 (Apolipoprotein A1), Apo-B (Apolipoprotein B), CHD (Coronary heart disease), CKD (Chronic kidney disease), DBP (Diastolic blood pressure), DCM (Dilated cardiomyopathy), FEV1 (Forced expiratory volume), FVC (Forced vital capacity), HDL-C (High-density lipoprotein cholesterol), HF (Heart failure), HbA1c (Haemoglobin A1c), LDL-C (Low-density lipoprotein cholesterol), LPa (Lipoprotein (a)), NAFLD (Non-alcoholic fatty liver disease), Non-ischemic CM (Non-ischemic cardiomyopathy), PBC (Primary biliary cirrhosis), PEF (Peak expiratory flow), SBP (Systolic blood pressure),SLE (Systemic lupus erythematosus), T2DM (Type 2 diabetes mellitus),

Trig (Triglycerides), VTE (Venous thromboembolism), eGFR (Estimated glomerular filtration rate)

Table S13: **Summary of all graph communities, clustered using Louvain community detection algorithm in Neo4j**. Enriched pathways identified by comparing the pathway membership of the proteins within the community against those outside the community. Enrichment formally evaluated by testing for the difference in proportions using a false-positive rate of 0.05. Abbreviations: AF (Atrial Fibrillation), ALS (Amyotrophic lateral sclerosis), AMD (Age-related macular degeneration), Apo-A1 (Apolipoprotein A1), Apo-B (Apolipoprotein B), CHD (Coronary heart disease), CKD (Chronic kidney disease), DBP (Diastolic blood pressure), DCM (Dilated cardiomyopathy), FEV1 (Forced expiratory volume), FVC (Forced vital capacity), HDL-C (High-density lipoprotein cholesterol), HF (Heart failure), HbA1c (Haemoglobin A1c), LDL-C (Low-density lipoprotein cholesterol), LPa (Lipoprotein (a)), NAFLD (Non-alcoholic fatty liver disease), Non-ischemic CM (Non-ischemic cardiomyopathy), PBC (Primary biliary cirrhosis), PEF (Peak expiratory flow), SBP (Systolic blood pressure),SLE (Systemic lupus erythematosus), T2DM (Type 2 diabetes mellitus), Trig (Triglycerides), VTE (Venous thromboembolism), eGFR (Estimated glomerular filtration rate).

Table S14: **Replication analysis results for all significant MR associations**. Results in the original analyses were evaluated for significance against a Bonferroni corrected p-value threshold of 5.16×10-6 based on the number of proteins and outcomes (Table S1). Analysis model used is either inverse variance weighted (IVW) or MR-Egger, as selected by the Rücker model selection framework. An association was considered replicated first if it reached a p-value threshold of 0.05 in the replication analysis and the effect direction was concordant with the original analysis. Each replicated association was then evaluated for replication using a Bonferroni corrected p-value threshold (6.85×10-4) based on the number of proteins analysed in the replication analysis (73 proteins). Effect estimates are reported as odds ratios. Abbreviations: AF (Atrial Fibrillation), ALS (Amyotrophic lateral sclerosis), AMD (Age-related macular degeneration), Apo-A1 (Apolipoprotein A1), Apo-B (Apolipoprotein B), CHD (Coronary heart disease), CKD (Chronic kidney disease), DBP (Diastolic blood pressure), DCM (Dilated cardiomyopathy), FEV1 (Forced expiratory volume), FVC (Forced vital capacity), HDL-C (High-density lipoprotein cholesterol), HF (Heart failure), HbA1c (Haemoglobin A1c), LDL-C (Low-density lipoprotein cholesterol), LPa (Lipoprotein (a)), NAFLD (Non-alcoholic fatty liver disease), Non-ischemic CM (Non-ischemic cardiomyopathy), PBC (Primary biliary cirrhosis), PEF (Peak expiratory flow), SBP (Systolic blood pressure),SLE (Systemic lupus erythematosus), T2DM (Type 2 diabetes mellitus), Trig (Triglycerides), VTE (Venous thromboembolism), eGFR (Estimated glomerular filtration rate).

Table S15: **Colocalisation analysis results for all significant MR associations**. Colocalisation was evaluated using a method adapted from Giambartolomei et al, and later updated by Wallace et al. A colocalisation event was defined as a probability of H4 (shared causal variant) of 0.8 or greater. Abbreviations: AF (Atrial Fibrillation), ALS (Amyotrophic lateral sclerosis), AMD (Age-related macular degeneration), Apo-A1 (Apolipoprotein A1), Apo-B (Apolipoprotein B), CHD (Coronary heart disease), CKD (Chronic kidney disease), DBP (Diastolic blood pressure), DCM (Dilated cardiomyopathy), FEV1 (Forced expiratory volume), FVC (Forced vital capacity), HDL-C (High-density lipoprotein cholesterol), HF (Heart failure), HbA1c (Haemoglobin A1c), LDL-C (Low-density lipoprotein cholesterol), LPa (Lipoprotein (a)), NAFLD (Non-alcoholic fatty liver disease), Non-ischemic CM (Non-ischemic cardiomyopathy), PBC (Primary biliary cirrhosis), PEF (Peak expiratory flow), SBP (Systolic blood pressure),SLE (Systemic lupus erythematosus), T2DM (Type 2 diabetes mellitus), Trig (Triglycerides), VTE (Venous thromboembolism), eGFR (Estimated glomerular filtration rate).

Table S16: **Associations with overlap between MR significance, replication and colocalisation evidence.** Results in the original analyses were evaluated for significance against a Bonferroni corrected p-value threshold of 5.16×10-6 based on the number of proteins and outcomes (Table S1). An association was considered to have replication and colocalisation evidence if it reached a p-value threshold of 0.05 in the replication analysis and the effect direction was concordant with the original analysis (Table S12), and had a probability of H4 (shared causal variant) of 0.8 or greater in the colocalisation analysis (Table S13). Abbreviations: AF (Atrial Fibrillation), ALS (Amyotrophic lateral sclerosis), AMD (Age-related macular degeneration), Apo-A1 (Apolipoprotein A1), Apo-B (Apolipoprotein B), CHD (Coronary heart disease), CKD (Chronic kidney disease), DBP (Diastolic blood pressure), DCM (Dilated cardiomyopathy), FEV1 (Forced expiratory volume), FVC (Forced vital capacity), HDL-C (High-density lipoprotein cholesterol), HF (Heart failure), HbA1c (Haemoglobin A1c), LDL-C (Low-density lipoprotein cholesterol), LPa (Lipoprotein (a)), NAFLD (Non-alcoholic fatty liver disease), Non-ischemic CM (Non-ischemic cardiomyopathy), PBC (Primary biliary cirrhosis), PEF (Peak expiratory flow), SBP (Systolic blood pressure),SLE (Systemic lupus erythematosus), T2DM (Type 2 diabetes mellitus), Trig (Triglycerides), VTE (Venous thromboembolism), eGFR (Estimated glomerular filtration rate)

Table S17: **Positive controls taken from ChEMBL and TrialTrove** for any protein-disease pair in the MR analyses that has a drug trialled at either phase 3 or phase 4.

Table S18: **All protein-quantitative trait loci (pQTLs) used in the Mendelian randomisation analysis.**

Table S19: **All proteins, alongside the pQTL study from which the data for each was taken, and the sample size of the study**. If a protein was available in more than one study then the study with the larger sample size was selected.

Table S20: **All outcomes with details of the corresponding genome-wide association study (GWAS)**. Abbreviations: AF (Atrial Fibrillation), ALS (Amyotrophic lateral sclerosis), AMD (Age-related macular degeneration), Apo-A1 (Apolipoprotein A1), Apo-B (Apolipoprotein B), CHD (Coronary heart disease), CKD (Chronic kidney disease), DBP (Diastolic blood pressure), DCM (Dilated cardiomyopathy), FEV1 (Forced expiratory volume), FVC (Forced vital capacity), HDL-C (High-density lipoprotein cholesterol), HF (Heart failure), HbA1c (Haemoglobin A1c), LDL-C (Low-density lipoprotein cholesterol), LPa (Lipoprotein (a)), NAFLD (Non-alcoholic fatty liver disease), Non-ischemic CM (Non-ischemic cardiomyopathy), PBC (Primary biliary cirrhosis), PEF (Peak expiratory flow), SBP (Systolic blood pressure),SLE (Systemic lupus erythematosus), T2DM (Type 2 diabetes mellitus), Trig (Triglycerides), VTE (Venous thromboembolism), eGFR (Estimated glomerular filtration rate)

Table S21: **All outcomes with assigned disease group and beneficial effect directions**. Biomarkers for which a plasma decrease is beneficial are labelled with 'decrease' and those for which a plasma increase is beneficial are labelled with 'increase'. All diseases have a beneficial direction of 'decrease'. All outcomes are categorised into one of 13 groups Abbreviations: AF (Atrial Fibrillation), ALS (Amyotrophic lateral sclerosis), AMD (Age-related macular degeneration), Apo-A1 (Apolipoprotein A1), Apo-B (Apolipoprotein B), CHD (Coronary heart disease), CKD (Chronic kidney disease), DBP (Diastolic blood pressure), DCM (Dilated cardiomyopathy), FEV1 (Forced expiratory volume), FVC (Forced vital capacity), HDL-C (High-density lipoprotein cholesterol), HF (Heart failure), HbA1c (Haemoglobin A1c), LDL-C (Low-density lipoprotein cholesterol), LPa (Lipoprotein (a)), NAFLD (Non-alcoholic fatty liver disease), Non-ischemic CM (Non-ischemic cardiomyopathy), PBC (Primary biliary cirrhosis), PEF (Peak expiratory flow), SBP (Systolic blood pressure),SLE (Systemic lupus erythematosus), T2DM (Type 2 diabetes mellitus), Trig (Triglycerides), VTE (Venous thromboembolism), eGFR (Estimated glomerular filtration rate).

Table S22: **Proteins with their interactors, as identified by the STRING database**.

## REFERENCES

1. C. Langenberg, A. D. Hingorani, C. J. M. Whitty, Biological and functional multimorbidity—From mechanisms to management. *Nat. Med.* **29**, 1649–1657 (2023).

2. V. Kuan, S. Denaxas, P. Patalay, D. Nitsch, R. Mathur, A. Gonzalez-Izquierdo, R. Sofat, L. Partridge, A. Roberts, I. C. K. Wong, M. Hingorani, N. Chaturvedi, H. Hemingway, A. D. Hingorani, D. C. Alexander, I. G. Asiimwe, S. Ball, F. Bennett, M. C. Borges, A. Butterworth, N. Chaturvedi, S. Chopade, C. Clarkson, M. Cox, C. Dale, S. Denaxas, D. Dunca, J. E. Engmann, A. Fernandez-Sanles, C. Finan, N. Fitzpatrick, J. Gallagher, A. Gonzalez-Izquierdo, J. Gratton, C. Gross, H. Hemingway, A. Henry, M. Hidajat, A. Hingorani, N. Hukerikar, A. Jorgensen, R. Joshi, M. Katsoulis, V. Kuan, R. Kumar, A. G. Lai, C. Langenberg, D. Lawlor, M. Mancini, D. Miller, M. Ogden, E. B. Ozyigit, S. Patel, M. Pirmohamed, A. Roberts, D. Ryan, A. F. Schmidt, A. D. Shah, T. Shah, R. Sofat, R. Takhar, A. Torralbo, A. Ullah, L. E. Walker, A. Warwick, E. Wheeler, V. L. Wright, H. Wu, M. Zwierzyna, Identifying and visualising multimorbidity and comorbidity patterns in patients in the English National Health Service: A population-based study. *Lancet Digit. Health* **5**, e16–e27 (2023).

3. A. D. Hingorani, V. Kuan, C. Finan, F. A. Kruger, A. Gaulton, S. Chopade, R. Sofat, R. J. MacAllister, J. P. Overington, H. Hemingway, S. Denaxas, D. Prieto, J. P. Casas, Improving the odds of drug development success through human genomics: Modelling study. *Sci. Rep.* **9**, 18911 (2019).

4. N. Hukerikar, A. D. Hingorani, F. W. Asselbergs, C. Finan, A. F. Schmidt, Prioritising genetic findings for drug target identification and validation. *Atherosclerosis* **390**, 117462 (2024).

5. S. Sivakumaran, F. Agakov, E. Theodoratou, J. G. Prendergast, L. Zgaga, T. Manolio, I. Rudan, P. M. Keigue, J. F. Wilson, H. Campbell, Abundant pleiotropy in human complex diseases and traits. *Am. J. Hum. Genet.* **89**, 607–618 2011.

6. R. Santos, O. Ursu, A. Gaulton, A. P. Bento, R. S. Donadi, C. G. Bologa, A. Karlsson, B. Al-Lazikani, A. Hersey, T. I. Oprea, J. P. Overington, A comprehensive map of molecular drug targets. *Nat. Rev. Drug Discov.* **16**, 19–34 (2017).

7. S. C. Harrison, A. J. P. Smith, G. T. Jones, D. I. Swerdlow, R. Rampuri, M. J. Bown, L. Folkersen, A. F. Baas, G. J. de Borst, J. D. Blankensteijn, J. F. Price, Y. van der Graaf, S. McLachlan, O. Agu, A. Hofman, A. G. Uitterlinden, A. Franco-Cereceda, Y. M. Ruigrok, F. N. van't Hof, J. T. Powell, A. M. van Rij, J. P. Casas, P. Eriksson, M. V. Holmes, F. W. Asselbergs, A. D. Hingorani, S. E. Humphries, Interleukin-6 receptor pathways in abdominal aortic aneurysm. *Eur. Heart J.* **34**, 3707–3716 (2013).

8. The Interleukin-6 Receptor Mendelian Randomisation Analysis (IL6R MR) Consortium, The interleukin-6 receptor as a target for prevention of coronary heart disease: A mendelian randomisation analysis. *Lancet* **379**, 1214–1224 (2012).

9. P. M. Ridker, B. M. Everett, T. Thuren, J. G. MacFadyen, W. H. Chang, C. Ballantyne, F. Fonseca, J. Nicolau, W. Koenig, S. D. Anker, J. J. P. Kastelein, J. H. Cornel, P. Pais, D. Pella, J. Genest, R. Cifkova, A. Lorenzatti, T. Forster, Z. Kobalava, L. Vida-Simiti, M. Flather, H. Shimokawa, H. Ogawa, M. Dellborg, P. R. F. Rossi, R. P. T. Troquay, P. Libby, R. J. Glynn, CANTOS Trial Group, Antiinflammatory therapy with canakinumab for atherosclerotic disease. *N. Engl. J. Med.* **377**, 1119–1131 (2017).

10. D. P. Wightman, I. E. Jansen, J. E. Savage, A. A. Shadrin, S. Bahrami, D. Holland, A. Rongve, S. Børte, B. S. Winsvold, O. K. Drange, A. E. Martinsen, A. H. Skogholt, C. Willer, G. Bråthen, I. Bosnes, J. B. Nielsen, L. G. Fritsche, L. F. Thomas, L. M. Pedersen, M. E. Gabrielsen, M. B. Johnsen, T. W. Meisingset, W. Zhou, P. Proitsi, A. Hodges, R. Dobson, L. Velayudhan, K. Heilbron, A. Auton, J. M. Sealock, L. K. Davis, N. L. Pedersen, C. A. Reynolds, I. K. Karlsson, S. Magnusson, H. Stefansson, S. Thordardottir, P. V. Jonsson, J. Snaedal, A. Zettergren, I. Skoog, S. Kern, M. Waern, H. Zetterberg, K. Blennow, E. Stordal, K. Hveem, J.-A. Zwart, L. Athanasiu, P. Selnes, I. Saltvedt, S. B. Sando, I. Ulstein, S. Djurovic, T. Fladby, D. Aarsland, G. Selbæk, S. Ripke, K. Stefansson, O. A. Andreassen, D. Posthuma, A genome-wide association study with 1,126,563 individuals identifies new risk loci for Alzheimer's disease. *Nat. Genet.* **53**, 1276–1282 (2021).

11. K. M. Fulton, S. M. Twine, "Immunoproteomics: Current technology and applications," in *Immunoproteomics: Methods and Protocols*, K. M. Fulton, S. M. Twine, Eds. (Humana Press, 2013), pp. 21–57.

12. I. B. McInnes, E. M. Gravallese, Immune-mediated inflammatory disease therapeutics: Past, present and future. *Nat. Rev. Immunol.* **21**, 680–686 (2021).

13. L. Castelo-Soccio, H. Kim, M. Gadina, P. L. Schwartzberg, A. Laurence, J. J. O'Shea, Protein kinases: Drug targets for immunological disorders. *Nat. Rev. Immunol.* **23**, 787–806 (2023).

14. D. I. Swerdlow, K. B. Kuchenbaecker, S. Shah, R. Sofat, M. V. Holmes, J. White, J. S. Mindell, M. Kivimaki, E. J. Brunner, J. C. Whittaker, J. P. Casas, A. D. Hingorani, Selecting instruments for Mendelian randomization in the wake of genome-wide association studies. *Int. J. Epidemiol.* **45**, 1600–1616 (2016).

15. A. F. Schmidt, A. D. Hingorani, C. Finan, Human genomics and drug development. *Cold Spring Harb. Perspect. Med.* **12**, a039230 (2022).

16. A. F. Schmidt, C. Finan, M. Gordillo-Marañón, F. W. Asselbergs, D. F. Freitag, R. S. Patel, B. Tyl, S. Chopade, R. Faraway, M. Zwierzyna, A. D. Hingorani, Genetic drug target validation using Mendelian randomisation. *Nat. Commun.* **11**, 3255 (2020).

17. A. F. Schmidt, N. B. Hunt, M. Gordillo-Marañón, P. Charoen, F. Drenos, M. Kivimaki, D. A. Lawlor, C. Giambartolomei, O. Papacosta, N. Chaturvedi, J. C. Bis, C. J. O'Donnell, G. Wannamethee, A. Wong, J. F. Price, A. D. Hughes, T. R. Gaunt, N. Franceschini, D. O. Mook-Kanamori, M. Zwierzyna, R. Sofat, A. D. Hingorani, C. Finan, Cholesteryl ester transfer protein (CETP) as a drug target for cardiovascular disease. *Nat. Commun.* **12**, 5640 (2021).

18. E. V. Minikel, J. L. Painter, C. C. Dong, M. R. Nelson, Refining the impact of genetic evidence on clinical success. *Nature* **629**, 624–629 (2024).

19. M. Gordillo-Marañón, A. F. Schmidt, A. Warwick, C. Tomlinson, C. Ytsma, J. Engmann, A. Torralbo, R. Maclean, R. Sofat, C. Langenberg, A. D. Shah, S. Denaxas, M. Pirmohamed, H.

Hemingway, A. D. Hingorani, C. Finan, Disease coverage of human genome-wide association studies and pharmaceutical research and development. *Commun. Med.* **4**, 195 (2024).

20. T. J. Lupancu, M. Eivazitork, J. A. Hamilton, A. A. Achuthan, K. M.-C. Lee, CCL17/TARC in autoimmunity and inflammation—Not just a T-cell chemokine. *Immunol. Cell Biol.* **101**, 600–609 (2023).

21. Y. Zhang, X. Tang, Z. Wang, L. Wang, Z. Chen, J.-Y. Qian, Z. Tian, S.-Y. Zhang, The chemokine CCL17 is a novel therapeutic target for cardiovascular aging. *Signal Transduct. Target. Ther.* **8**, 157 (2023).

22. M. Del Mar Maldonado, J. Schlom, D. H. Hamilton, Blockade of tumor-derived colony-stimulating factor 1 (CSF1) promotes an immune-permissive tumor microenvironment. *Cancer Immunol. Immunother.* **72**, 3349–3362 (2023).

23. D. A. Hume, K. P. A. MacDonald, Therapeutic applications of macrophage colony-stimulating factor-1 (CSF-1) and antagonists of CSF-1 receptor (CSF-1R) signaling. *Blood* **119**, 1810–1820 (2012).

24. J. Muñoz-Garcia, D. Cochonneau, S. Télétchéa, E. Moranton, D. Lanoe, R. Brion, F. Lézot, M.-F. Heymann, D. Heymann, The twin cytokines interleukin-34 and CSF-1: Masterful conductors of macrophage homeostasis. *Theranostics* **11**, 1568–1593 (2021).

25. P. A. Gerlach, N. Milind, J. P. Spence, J. K. Pritchard, High false sign rates in transcriptome-wide association studies. bioRxiv 695550 [Preprint] (2025); https://doi.org/10.64898/2025.12.19.695550.

26. L. Su, Y. Wang, J. Wang, Y. Mifune, M. D. Morin, B. T. Jones, E. M. Y. Moresco, D. L. Boger, B. Beutler, H. Zhang, Structural basis of TLR2/TLR1 activation by the synthetic agonist diprovocim. *J. Med. Chem.* **62**, 2938–2949 (2019).

27. M. Luchner, S. Reinke, A. Milicic, TLR agonists as vaccine adjuvants targeting cancer and infectious diseases. *Pharmaceutics* **13**, 142 (2021).

28. H. Communications, “Zinbryta withdrawn from marketplace,” *MSAA* (2018); https://mymsaa.org/news/zinbryta-withdrawn/.

29. “Daclizumab withdrawn from NHS after safety concerns,” *MS Society* (2018); www.mssociety.org.uk/research/news/daclizumab-withdrawn-nhs-after-safety-concerns.

30. J. Boulet, V. S. Sridhar, N. Bouabdallaoui, J.-C. Tardif, M. White, Inflammation in heart failure: Pathophysiology and therapeutic strategies. *Inflamm. Res.* **73**, 709–723 (2024).

31. D. Harding, M. H. A. Chong, N. Lahoti, C. M. Bigogno, R. Prema, S. A. Mohiddin, F. Marelli-Berg, Dilated cardiomyopathy and chronic cardiac inflammation: Pathogenesis, diagnosis and therapy. *J. Intern. Med.* **293**, 23–47 (2023).

32. C. Novoa, P. Salazar, P. Cisternas, C. Gherardelli, R. Vera-Salazar, J. M. Zolezzi, N. C. Inestrosa, Inflammation context in Alzheimer’s disease, a relationship intricate to define. *Biol. Res.* **55**, 39 (2022).

33. P. Zhou, Emerging mechanisms and applications of low-dose IL-2 therapy in autoimmunity. *Cytokine Growth Factor Rev.* **67**, 80–88 (2022).

34. D. Dermawan, N. Alotaiq, Computational analysis of antimicrobial peptides targeting key receptors in infection-related cardiovascular diseases: Molecular docking and dynamics insights. *Sci. Rep.* **15**, 8896 (2025).

35. Zenas BioPharma (USA) LLC, “A phase 3, multicenter, randomized, double-blind, placebo-controlled study with an open label safety and dose confirmation run-in period, to evaluate the efficacy and safety of obexelimab in patients with warm autoimmune hemolytic anemia (SApHiAre)” (Clinical trial registration NCT05786573, clinicaltrials.gov, 2025); https://clinicaltrials.gov/study/NCT05786573.

36. Zenas BioPharma (USA) LLC, “A phase 3, multicenter, randomized, double-blind, placebo-controlled study to evaluate the efficacy and safety of obexelimab in patients with IgG4-related disease (INDIGO)” (Clinical trial registration NCT05662241, clinicaltrials.gov, 2024); https://clinicaltrials.gov/study/NCT05662241.

37. J. T. Merrill, J. Guthridge, M. Smith, J. June, F. Koumpouras, W. Machua, A. Askanase, A. Khosroshahi, S. Z. Sheikh, G. Rathi, B. Burington, P. Foster, M. Matijevic, S. Arora, X. Wang, M. Gao, S. Wax, J. A. James, D. J. Zack, Obexelimab in systemic lupus erythematosus with exploration of response based on gene pathway co-expression patterns: A double-blind, randomized, placebo-controlled, phase 2 trial. *Arthritis Rheumatol.* **75**, 2185–2194 (2023).

38. S. Aboobacker, H. Kurn, A. M. Al Aboud, "Secukinumab," in *StatPearls* (StatPearls Publishing, 2025); http://www.ncbi.nlm.nih.gov/books/NBK537091/.

39. A. K. Greda, J. P. Gomes, V. Schmidt-Krueger, E. Zurawska-Plaksej, R. Fritsche-Guenther, I.-M. Rudolph, N. S. Telugu, C. Cömert, J. Kirwan, S. Kunz, M. Rothe, M. Johannsen, S. Diecke, P. Bross, T. E. Willnow, Interaction of sortilin with apolipoprotein E3 enables neurons to use long-chain fatty acids as alternative metabolic fuel. *Nat. Metab.* **7**, 2346–2365 (2025).

40. The UniProt Consortium, UniProt: The universal protein knowledgebase in 2021. *Nucleic Acids Res.* **49**, D480–D489 (2021).

41. E. B. Fauman, C. Hyde, An optimal variant to gene distance window derived from an empirical definition of cis and trans protein QTLs. *BMC Bioinformatics* **23**, 169 (2022).

42. S. Burgess, V. Zuber, E. Valdes-Marquez, B. B. Sun, J. C. Hopewell, Mendelian randomization with fine-mapped genetic data: Choosing from large numbers of correlated instrumental variables. *Genet. Epidemiol.* **41**, 714–725 (2017).

43. C. Sudlow, J. Gallacher, N. Allen, V. Beral, P. Burton, J. Danesh, P. Downey, P. Elliott, J. Green, M. Landray, B. Liu, P. Matthews, G. Ong, J. Pell, A. Silman, A. Young, T. Sprosen, T. Peakman, R. Collins, UK Biobank: An open access resource for identifying the causes of a wide range of complex diseases of middle and old age. *PLoS Med.* **12**, e1001779 (2015).

44. J. Bowden, G. Davey Smith, P. C. Haycock, S. Burgess, Consistent estimation in Mendelian randomization with some invalid instruments using a weighted median estimator. *Genet. Epidemiol.* **40**, 304–314 (2016).

45. J. Bowden, G. Davey Smith, S. Burgess, Mendelian randomization with invalid instruments: Effect estimation and bias detection through Egger regression. *Int. J. Epidemiol.* **44**, 512–525 (2015).

46. J. Bowden, F. Del Greco M., C. Minelli, G. D. Smith, N. Sheehan, J. Thompson, A framework for the investigation of pleiotropy in two-sample summary data Mendelian randomization. *Stat. Med.* **36**, 1783–1802 (2017).

47. D. A. Lawlor, R. M. Harbord, J. A. C. Sterne, N. Timpson, G. Davey Smith, Mendelian randomization: Using genes as instruments for making causal inferences in epidemiology. *Stat. Med.* **27**, 1133–1163 (2008).

48. E. Sjöstedt, W. Zhong, L. Fagerberg, M. Karlsson, N. Mitsios, C. Adori, P. Oksvold, F. Edfors, A. Limiszewska, F. Hikmet, J. Huang, Y. Du, L. Lin, Z. Dong, L. Yang, X. Liu, H. Jiang, X. Xu, J. Wang, H. Yang, L. Bolund, A. Mardinoglu, C. Zhang, K. von Feilitzen, C. Lindskog, F. Pontén, Y. Luo, T. Hökfelt, M. Uhlén, J. Mulder, An atlas of the protein-coding genes in the human, pig, and mouse brain. *Science* **367**, eaay5947 (2020).

49. The Human Protein Atlas; www.proteinatlas.org/.

50. K. Lage, N. T. Hansen, E. O. Karlberg, A. C. Eklund, F. S. Roque, P. K. Donahoe, Z. Szallasi, T. S. Jensen, S. Brunak, A large-scale analysis of tissue-specific pathology and gene expression of human disease genes and complexes. *Proc. Natl. Acad. Sci. U.S.A.* **105**, 20870–20875 (2008).

51. J. White, PubMed 2.0. *Med. Ref. Serv. Q.* **39**, 382–387 (2020).

52. M. Gremse, A. Chang, I. Schomburg, A. Grote, M. Scheer, C. Ebeling, D. Schomburg, The BRENDA Tissue Ontology (BTO): The first all-integrating ontology of all organisms for enzyme sources. *Nucleic Acids Res.* **39**, D507–D513 (2011).

53. O. Bodenreider, The Unified Medical Language System (UMLS): Integrating biomedical terminology. *Nucleic Acids Res.* **32**, D267–D270 (2004).

54. C. Finan, A. Gaulton, Felix. A. Kruger, R. T. Lumbers, T. Shah, J. Engmann, L. Galver, R. Kelley, A. Karlsson, R. Santos, J. P. Overington, A. D. Hingorani, J. P. Casas, The druggable genome and support for target identification and validation in drug development. *Sci. Transl. Med.* **9**, eaag1166 (2017).

55. A. Gaulton, L. J. Bellis, A. P. Bento, J. Chambers, M. Davies, A. Hersey, Y. Light, S. McGlinchey, D. Michalovich, B. Al-Lazikani, J. P. Overington, ChEMBL: A large-scale bioactivity database for drug discovery. *Nucleic Acids Res.* **40**, D1100–D1107 (2012).

56. Clinical Trial Research | Trialtrove l Pharma Intelligence; https://pharmaintelligence.informa.com/products-and-services/clinical-planning/trialtrove.

57. C. von Mering, M. Huynen, D. Jaeggi, S. Schmidt, P. Bork, B. Snel, STRING: A database of predicted functional associations between proteins. *Nucleic Acids Res.* **31**, 258–261 (2003).

58. A. Fabregat, F. Korninger, G. Viteri, K. Sidiropoulos, P. Marin-Garcia, P. Ping, G. Wu, L. Stein, P. D'Eustachio, H. Hermjakob, Reactome graph database: Efficient access to complex pathway data. *PLoS Comput. Biol.* **14**, e1005968 (2018).

59. S. A. Aleksander, J. Balhoff, S. Carbon, J. M. Cherry, H. J. Drabkin, D. Ebert, M. Feuermann, P. Gaudet, N. L. Harris, D. P. Hill, R. Lee, H. Mi, S. Moxon, C. J. Mungall, A. Muruganugan, T. Mushayahama, P. W. Sternberg, P. D. Thomas, K. Van Auken, J. Ramsey, D. A. Siegele, R. L. Chisholm, P. Fey, M. C. Aspromonte, M. V. Nugnes, F. Quaglia, S. Tosatto, M. Giglio, S. Nadendla, G. Antonazzo, H. Attrill, G. dos Santos, S. Marygold, V. Strelets, C. J. Tabone, J. Thurmond, P. Zhou, S. H. Ahmed, P. Asanitthong, D. Luna Buitrago, M. N. Erdol, M. C. Gage, M. Ali Kadhum, K. Y. C. Li, M. Long, A. Michalak, A. Pesala, A. Pritazahra, S. C. C. Saverimuttu, R. Su, K. E. Thurlow, R. C. Lovering, C. Logie, S. Oliferenko, J. Blake, K. Christie, L. Corbani, M. E. Dolan, H. J. Drabkin, D. P. Hill, L. Ni, D. Sitnikov, C. Smith, A. Cuzick, J. Seager, L. Cooper, J. Elser, P. Jaiswal, P. Gupta, P. Jaiswal, S. Naithani, M. Lera-Ramirez, K. Rutherford, V. Wood, J. L. De Pons, M. R. Dwinell, G. T. Hayman, M. L. Kaldunski, A. E. Kwitek, S. J. F. Laulederkind, M. A. Tutaj, M. Vedi, S.-J. Wang, P. D'Eustachio, L. Aimo, K. Axelsen, A. Bridge, N. Hyka-Nouspikel, A. Morgat, S. A. Aleksander, J. M. Cherry, S. R. Engel, K. Karra, S. R. Miyasato, R. S. Nash, M. S. Skrzypek, S. Weng, E. D.

Wong, E. Bakker, T. Z. Berardini, L. Reiser, A. Auchincloss, K. Axelsen, G. Argoud-Puy, M.-C. Blatter, E. Boutet, L. Breuza, A. Bridge, C. Casals-Casas, E. Coudert, A. Estreicher, M. Livia Famiglietti, M. Feuermann, A. Gos, N. Gruaz-Gumowski, C. Hulo, N. Hyka-Nouspikel, F. Jungo, P. Le Mercier, D. Lieberherr, P. Masson, A. Morgat, I. Pedruzzi, L. Pourcel, S. Poux, C. Rivoire, S. Sundaram, A. Bateman, E. Bowler-Barnett, H. Bye-A-Jee, P. Denny, A. Ignatchenko, R. Ishtiaq, A. Lock, Y. Lussi, M. Magrane, M. J. Martin, S. Orchard, P. Raposo, E. Speretta, N. Tyagi, K. Warner, R. Zaru, A. D. Diehl, R. Lee, J. Chan, S. Diamantakis, D. Raciti, M. Zarowiecki, M. Fisher, C. James-Zorn, V. Ponferrada, A. Zorn, S. Ramachandran, L. Ruzicka, M. Westerfield, The gene ontology knowledgebase in 2023. *Genetics* **224**, iyad031 (2023).

60. Neo4j Graph Data Platform–The Leader in Graph Databases, *Neo4j Graph Data Platform*; https://neo4j.com/.

61. I. K. Dhammi, S. Kumar, Medical subject headings (MeSH) terms. *Indian J. Orthop.* **48**, 443–444 (2014).

62. J. Hoppa, Graph Algorithms in Neo4j: Louvain Modularity, *Graph Database & Analytics* (2019): https://neo4j.com/blog/graph-algorithms-neo4j-louvain-modularity/.

63. B. B. Sun, J. Chiou, M. Traylor, C. Benner, Y.-H. Hsu, T. G. Richardson, P. Surendran, A. Mahajan, C. Robins, S. G. Vasquez-Grinnell, L. Hou, E. M. Kvikstad, O. S. Burren, J. Davitte, K. L. Ferber, C. E. Gillies, Å. K. Hedman, S. Hu, T. Lin, R. Mikkilineni, R. K. Pendergrass, C. Pickering, B. Prins, D. Baird, C.-Y. Chen, L. D. Ward, A. M. Deaton, S. Welsh, C. M. Willis, N. Lehner, M. Arnold, M. A. Wörheide, K. Suhre, G. Kastenmüller, A. Sethi, M. Cule, A. Raj, H. M. Kang, L. Burkitt-Gray, E. Melamud, M. H. Black, E. B. Fauman, J. M. M. Howson, H. M. Kang, M. I. McCarthy, P. Nioi, S. Petrovski, R. A. Scott, E. N. Smith, S. Szalma, D. M. Waterworth, L. J. Mitnaul, J. D. Szustakowski, B. W. Gibson, M. R. Miller, C. D. Whelan, Plasma proteomic associations with genetics and health in the UK Biobank. *Nature* **622**, 329–338 (2023).

64. C. Giambartolomei, D. Vukcevic, E. E. Schadt, L. Franke, A. D. Hingorani, C. Wallace, V. Plagnol, Bayesian test for colocalisation between pairs of genetic association studies using summary statistics. *PLOS Genet.* **10**, e1004383 (2014).

65. C. Wallace, A more accurate method for colocalisation analysis allowing for multiple causal variants. *PLOS Genet.* **17**, e1009440 (2021).

66. S. Said, R. Pazoki, V. Karhunen, U. Võsa, S. Ligthart, B. Bodinier, F. Koskeridis, P. Welsh, B. Z. Alizadeh, D. I. Chasman, N. Sattar, M. Chadeau-Hyam, E. Evangelou, M.-R. Jarvelin, P. Elliott, I. Tzoulaki, A. Dehghan, Genetic analysis of over half a million people characterises C-reactive protein loci. *Nat. Commun.* **13**, 2198 (2022).

67. E. Ferkingstad, P. Sulem, B. A. Atlason, G. Sveinbjornsson, M. I. Magnusson, E. L. Styrmisdottir, K. Gunnarsdottir, A. Helgason, A. Oddsson, B. V. Halldorsson, B. O. Jensson, F. Zink, G. H. Halldorsson, G. Masson, G. A. Arnadottir, H. Katrinardottir, K. Juliusson, M. K. Magnusson, O. T. Magnusson, R. Fridriksdottir, S. Saevarsdottir, S. A. Gudjonsson, S. N. Stacey, S. Rognvaldsson, T. Eiriksdottir, T. A. Olafsdottir, V. Steinthorsdottir, V. Tragante, M. O. Ulfarsson, H. Stefansson, I. Jonsdottir, H. Holm, T. Rafnar, P. Melsted, J. Saemundsdottir, G. L. Norddahl, S. H. Lund, D. F. Gudbjartsson, U. Thorsteinsdottir, K. Stefansson, Large-scale integration of the plasma proteome with genetics and disease. *Nat. Genet.* **53**, 1712–1721 (2021).

68. A. Gilly, Y.-C. Park, G. Png, A. Barysenka, I. Fischer, T. Bjørnland, L. Southam, D. Suveges, S. Neumeyer, N. W. Rayner, E. Tsafantakis, M. Karaleftheri, G. Dedoussis, E. Zeggini, Whole-genome sequencing analysis of the cardiometabolic proteome. *Nat. Commun.* **11**, 6336 (2020).

69. B. B. Sun, J. C. Maranville, J. E. Peters, D. Stacey, J. R. Staley, J. Blackshaw, S. Burgess, T. Jiang, E. Paige, P. Surendran, C. Oliver-Williams, M. A. Kamat, B. P. Prins, S. K. Wilcox, E. S. Zimmerman, A. Chi, N. Bansal, S. L. Spain, A. M. Wood, N. W. Morrell, J. R. Bradley, N. Janjic, D. J. Roberts, W. H. Ouwehand, J. A. Todd, N. Soranzo, K. Suhre, D. S. Paul, C. S. Fox, R. M. Plenge, J. Danesh, H. Runz, A. S. Butterworth, Genomic atlas of the human plasma proteome. *Nature* **558**, 73–79 (2018).

70. L. Folkersen, S. Gustafsson, Q. Wang, D. H. Hansen, Å. K. Hedman, A. Schork, K. Page, D. V. Zhernakova, Y. Wu, J. Peters, N. Eriksson, S. E. Bergen, T. Boutin, A. D. Bretherick, S. Enroth, A. Kalnapenkis, J. R. Gådin, B. Suur, Y. Chen, L. Matic, J. D. Gale, J. Lee, W. Zhang, A. Quazi, M. Ala-Korpela, S. H. Choi, A. Claringbould, J. Danesh, G. Davey-Smith, F. de Masi, S. Elmståhl, G. Engström, E. Fauman, C. Fernandez, L. Franke, P. Franks, V. Giedraitis, C. Haley,

A. Hamsten, A. Ingason, Å. Johansson, P. K. Joshi, L. Lind, C. M. Lindgren, S. Lubitz, T. Palmer, E. Macdonald-Dunlop, M. Magnusson, O. Melander, K. Michaelsson, A. P. Morris, R. Mägi, M. W. Nagle, P. M. Nilsson, J. Nilsson, M. Orho-Melander, O. Polasek, B. Prins, E. Pålsson, T. Qi, M. Sjögren, J. Sundström, P. Surendran, U. Võsa, T. Werge, R. Wernersson, H.-J. Westra, J. Yang, A. Zhernakova, J. Ärnlöv, J. Fu, G. Smith, T. Esko, C. Hayward, U. Gyllensten, M. Landen, A. Siegbahn, J. F. Wilson, L. Wallentin, A. S. Butterworth, M. V. Holmes, E. Ingelsson, A. Mälarstig, Genomic and drug target evaluation of 90 cardiovascular proteins in 30,931 individuals. *Nat. Metab.* **2**, 1135–1148 (2020).

71. C. Yang, F. H. G. Farias, L. Ibanez, A. Suhy, B. Sadler, M. V. Fernandez, F. Wang, J. L. Bradley, B. Eiffert, J. A. Bahena, J. P. Budde, Z. Li, U. Dube, Y. J. Sung, K. A. Mihindukulasuriya, J. C. Morris, A. M. Fagan, R. J. Perrin, B. A. Benitez, H. Rhinn, O. Harari, C. Cruchaga, Genomic atlas of the proteome from brain, CSF and plasma prioritizes proteins implicated in neurological disorders. *Nat. Neurosci.* **24**, 1302–1312 (2021).

72. C. Yao, G. Chen, C. Song, J. Keefe, M. Mendelson, T. Huan, B. B. Sun, A. Laser, J. C. Maranville, H. Wu, J. E. Ho, P. Courchesne, A. Lyass, M. G. Larson, C. Gieger, J. Graumann, A. D. Johnson, J. Danesh, H. Runz, S.-J. Hwang, C. Liu, A. S. Butterworth, K. Suhre, D. Levy, Genome-wide mapping of plasma protein QTLs identifies putatively causal genes and pathways for cardiovascular disease. *Nat. Commun.* **9**, 3268 (2018).

73. A. V. Ahola-Olli, P. Würtz, A. S. Havulinna, K. Aalto, N. Pitkänen, T. Lehtimäki, M. Kähönen, L.-P. Lyytikäinen, E. Raitoharju, I. Seppälä, A.-P. Sarin, S. Ripatti, A. Palotie, M. Perola, J. S. Viikari, S. Jalkanen, M. Maksimow, V. Salomaa, M. Salmi, J. Kettunen, O. T. Raitakari, Genome-wide association study identifies 27 loci influencing concentrations of circulating cytokines and growth factors. *Am. J. Hum. Genet.* **100**, 40–50 (2017).

74. N. Kryuchkova-Mostacci, M. Robinson-Rechavi, A benchmark of gene expression tissue-specificity metrics. *Brief. Bioinform.* **18**, 205–214 (2017).
